# Aggregation-Induced Emission-Active Photosensitizer-Mediated Photodynamic Therapy for Anti-Psoriasis

**DOI:** 10.34133/research.0344

**Published:** 2024-06-06

**Authors:** Ping Zhu, Zhaoji Wu, Zhilu Yang, Tingting Tang, Yunhui Liao, Wen Zhao, Ying Huang, Tao Chen, Junjie Li, Chunmei Nong, Zhenzhen Wu, Guodong Hu, Yanshan Liu, Yinghua Chen

**Affiliations:** ^1^ Department of Histology and Embryology, NMPA Key Laboratory for Safety Evaluation of Cosmetics, School of Basic Medical Sciences, Southern Medical University, Guangzhou 510515, China.; ^2^ Dongguan People’s Hospital Biobank, Affiliated Dongguan Hospital, Southern Medical University, Dongguan, Guangdong 523059, China.; ^3^ Guangdong Provincial Key Laboratory of Construction and Detection in Tissue Engineering, School of Basic Medical Sciences, Southern Medical University, Guangzhou 510515, China.; ^4^ NMPA Key Laboratory for Research and Evaluation of Drug Metabolism & Guangdong Provincial Key Laboratory of New Drug Screening, School of Pharmaceutical Sciences, Southern Medical University, Guangzhou 510515, China.; ^5^ Dongguan Key Laboratory of Smart Biomaterials and Regenerative Medicine, The Tenth Affiliated Hospital, Southern Medical University, Dongguan, Guangdong 523059, China.; ^6^ Department of Medical Imaging, Guangzhou Women and Children’s Medical Center, National Children’s Medical Center for South Central Region, Guangzhou 510515, China.; ^7^ Health Management Center, The Tenth Affiliated Hospital, Southern Medical University, Dongguan, Guangdong 523059, China.; ^8^ Nanfang Hospital Biobank, Clinical Research Center, Nanfang Hospital, Southern Medical University, No. 1838 Guangzhou Avenue, Guangzhou 510515, China.; ^9^ Department of Dermatology, The Tenth Affiliated Hospital, Southern Medical University, Dongguan, Guangdong 523059, China.; ^10^ Department of Respiratory and Critical Care Medicine, Dongguan Key Laboratory of Clinical Translation of Basic Research on Respiratory Diseases, The Tenth Affiliated Hospital, Southern Medical University, Dongguan, Guangdong 523059, China.

## Abstract

Hyperproliferative keratinocytes and subcutaneous inflammation contribute to the characteristic symptoms of psoriasis, including erythema, scales, or scaly plaques on the skin. These symptoms significantly affect patients’ quality of life and cause severe physical and psychological distress. However, current treatment strategies have limited therapeutic effect and may lead to adverse side effects. In this study, we present the novel organic photosensitizer TBTDC [5-(((5-(7-(4-(diphenylamino)phenyl)benzo[c][1,2,5]thiadiazol-4-yl)thiophen-2-yl)methylene)amino)-3-methylthiophene-2,4-dicarbonitrile] nanoparticles (NPs) with aggregation-induced emission (AIE) characteristics to mediate photodynamic therapy (TBTDC NP-PDT) for psoriasis treatment. We demonstrate that TBTDC NPs effectively generate reactive oxygen species upon light irradiation and lead to significant apoptosis of psoriatic keratinocytes. Furthermore, TBTDC NPs exhibit high cellular uptake in diseased keratinocytes and induce endoplasmic reticulum stress (ERS)-mediated autophagy, which can also enhance apoptosis. Importantly, TBTDC NPs show no cytotoxicity toward keratinocytes. These unique properties of TBTDC NPs enable remarkable therapeutic effects against psoriasis-like skin lesions and related inflammation in vivo. Overall, our AIE-active TBTDC NP-PDT represents a promising strategy for treating psoriasis in clinical settings.

## Introduction

Psoriasis is a chronic, hyperproliferative, and inflammatory skin disease that affects 2 to 3% of the population worldwide [1–3]. Typical symptoms of psoriasis include epidermal changes, such as erythema, scales, or scaly plaques on the skin [4,5], which cause great physical and psychological pain to patients, and seriously affect the quality of life [6,7]. Excessive proliferation and abnormal differentiation of keratinocytes, increased infiltration of related immune cells in the dermis, and release of inflammatory cytokines are the main pathological changes of psoriasis [8,9]. The stimulation of various inflammatory cytokines increases the proliferation of keratinocytes, and hyperproliferative keratinocytes produce many inflammatory cytokines that maintain and amplify the inflammatory response [9–11]. Hence, strategies capable of attenuating keratinocyte hyperproliferation and/or hyperinflammation have been suggested as potential psoriasis treatments.

Psoriasis is primarily treated with pharmacotherapy and physical therapy now, including the topical application of calcipotriol (CAL) drugs and photodynamic therapy (PDT) [12,13]. PDT represents an oxygen-dependent photochemical reaction based on the photochemical reaction that occurs among irradiation, a photosensitizer, and molecular oxygen. First, the photosensitizer accumulates in the target tissue and then absorbs light upon irradiation with a specific wavelength, which leads to its transition from ground state to excited singlet state and further development to triplet state with longer lifetime through intersystem crossing. This excited state can undergo two kinds of reactions. On the one hand, the triplet state can directly transfer energy to molecular oxygen leading to the generation of singlet oxygen, which is called type II reaction. On the other hand, it can react directly with substrates like cell membranes and molecules leading to the formation of free radicals and radical ions through electron transfer, which further react with molecular oxygen to produce O_2_^−•^, HO^•^, and H_2_O_2_, called type I reaction. Reactive oxygen species (ROS) produced by both reaction types can interact with a large number of biological substrates, which leads to the direct induction of oxidative damage and accelerated cell death [14–18]. The photosensitizer is the core of PDT and largely determines the effects [19–21]. However, traditional photosensitizers used in PDT have some inherent disadvantages, such as lower solubility in aqueous solutions, easy aggregation in concentrated solutions, and a tendency for emission quenching in the solid state. These characteristics result in reduced ROS generation and ineffective therapy [21,22]. The aggregation-induced emission (AIE) phenomenon was discovered by Tang in 2001 [23], and it is the opposite of the general aggregation-caused quenching effect observed for traditional luminophores at high concentrations or in the aggregated state. AIE-based photosensitizers exhibit enhanced ROS generation upon aggregation [24]. Currently, AIE materials are widely used in biomedicine, including bioimaging, cancer therapy, gene delivery, and antimicrobial applications [25–27]; nonetheless, the application of AIE to treat proliferative skin diseases has not yet been reported. We previously showed that TBTDC [5-(((5-(7-(4-(diphenylamino)phenyl)benzo[c][1,2,5]thiadiazol-4-yl)thiophen-2-yl)methylene)amino)-3-methylthiophene-2,4-dicarbonitrile] nanoparticles (NPs) are novel and highly efficient organic photosensitizers with AIE characteristics [28]. Compared with TBTDC, TBTDC NPs are more prone to cellular uptake and thus have broader biological application value; most importantly, our group has identified that TBTDC NP-PDT can effectively suppress tumor growth [28]. However, the effects of TBTDC NP-PDT on the hyperproliferative keratinocytes and abnormal inflammatory response in psoriasis remain unclear.

Photosensitizers with organellar targeting functions are recognized more effective in damaging cells, which is an excellent advantage of current photosensitizer materials [29]. When a photosensitizer enters the cell and is exposed to irradiation, it is excited from the ground state to an excited state and transfers energy to oxygen molecules, eventually leading to ROS generation and cellular damage [16]. Photosensitizers that produce large amounts of ROS under light irradiation can damage cells more effectively [30,31]. Some studies have shown that PDT promotes apoptosis by producing large amounts of ROS [32,33]. In recent years, a few studies have proposed that PDT can also promote autophagy, which may promote or inhibit PDT-induced apoptosis depending on the cell type and specific stimulant intensity [34,35].

In this study, AIE-active photosensitizer TBTDC NPs mediating PDT is applied in the treatment of psoriasis for the first time, which is proved to effectively ameliorate psoriasis-like skin lesions and psoriasis-related inflammation in vivo and in vitro. Moreover, we found that TBTDC NP-PDT could enhance apoptosis not only by generating large amounts of ROS but also through endoplasmic reticulum stress (ERS)-mediated autophagy from TBTDC NP targeting the endoplasmic reticulum in psoriasis keratinocytes. Our work provides insights into the development of novel and highly effective photosensitizers for psoriasis treatment.

## Results

### Characteristics and nontoxicity of TBTDC NPs

**Fig. 1.**
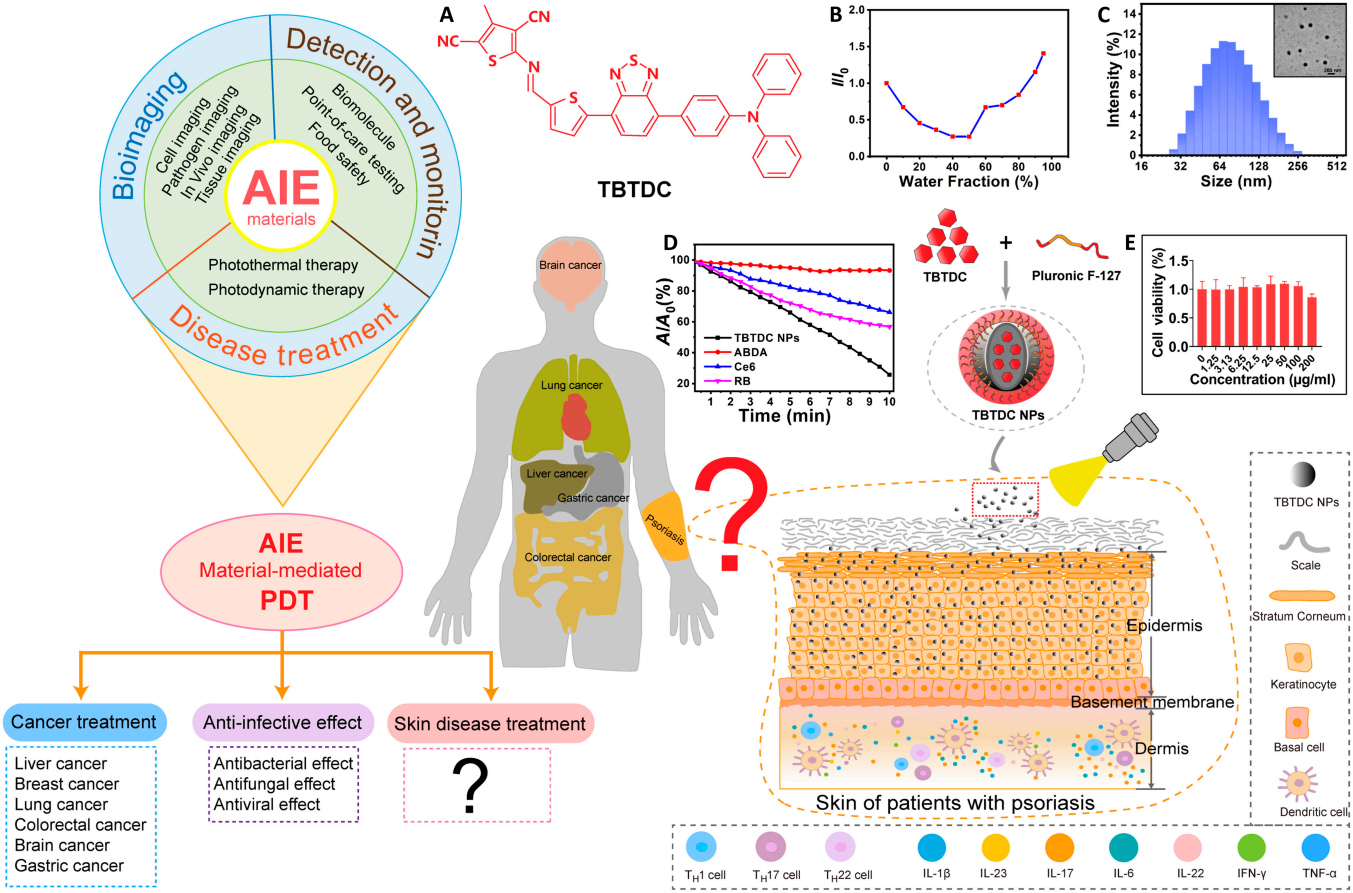
Chemical structure of TBTDC and characterization of TBTDC NPs. (A) Chemical structure of TBTDC. (B) Fluorescence intensity ratios (*I*/*I*_0_) of TBTDC in THF/water mixtures with different *f*_w_s at the maximum emission wavelength. *I*_0_ is the fluorescence intensity of TBTDC in THF. (C) Size distribution and TEM image of TBTDC NPs. (D) Decomposition of ABDA in the presence of TBTDC NPs, Ce6, or RB upon irradiation, where *A*_0_ and *A* are the absorbance of ABDA at 378 nm before and after irradiation, respectively. (E) Viability of HaCaT cells treated with different concentrations of TBTDC NPs for 24 h using the CCK-8 method.

The chemical structure of TBTDC is shown in Fig. 1A, and the AIE features of TBTDC are shown in Fig. S1B and Fig. 1B. To further explore the bioapplications of TBTDC, TBTDC NPs were prepared as described in our previous study [28]. As shown in Fig. S1C and D, TBTDC NPs had a wide absorption range up to 750 nm and a maximum absorption wavelength of 525 nm, and the emission maximum of them in tetrahydrofuran (THF) was 825 nm. The hydrodynamic diameter of TBTDC NPs is ~74 nm, and the polydispersity is ~0.17 (Fig. S1E and Fig. 1C). In addition, TBTDC NPs exhibited high ^1^O_2_ production efficiency compared to Chlorin e6 (Ce6) and Rose Bengal (RB) (Fig. S1F to I and Fig. 1D). Our previous studies showed that TBTDC NPs exhibit low cytotoxicity in HeLa, HepG2, MCF-7, and MCF-10 cells [28]. In this experiment, no obvious cytotoxicity was also found in human immortalized epidermal cells (HaCaT) incubated with TBTDC NPs, even at concentrations as high as 200 μg/ml for 24 h, compared with that of the HaCaT cell group according to the Cell Counting Kit-8 (CCK-8) assay (Fig. 1H). These results indicate that TBTDC NPs have low cytotoxicity and good biocompatibility.

### TBTDC NP-PDT ameliorated imiquimod-induced psoriasis-like skin lesions in mice

**Fig. 2.**
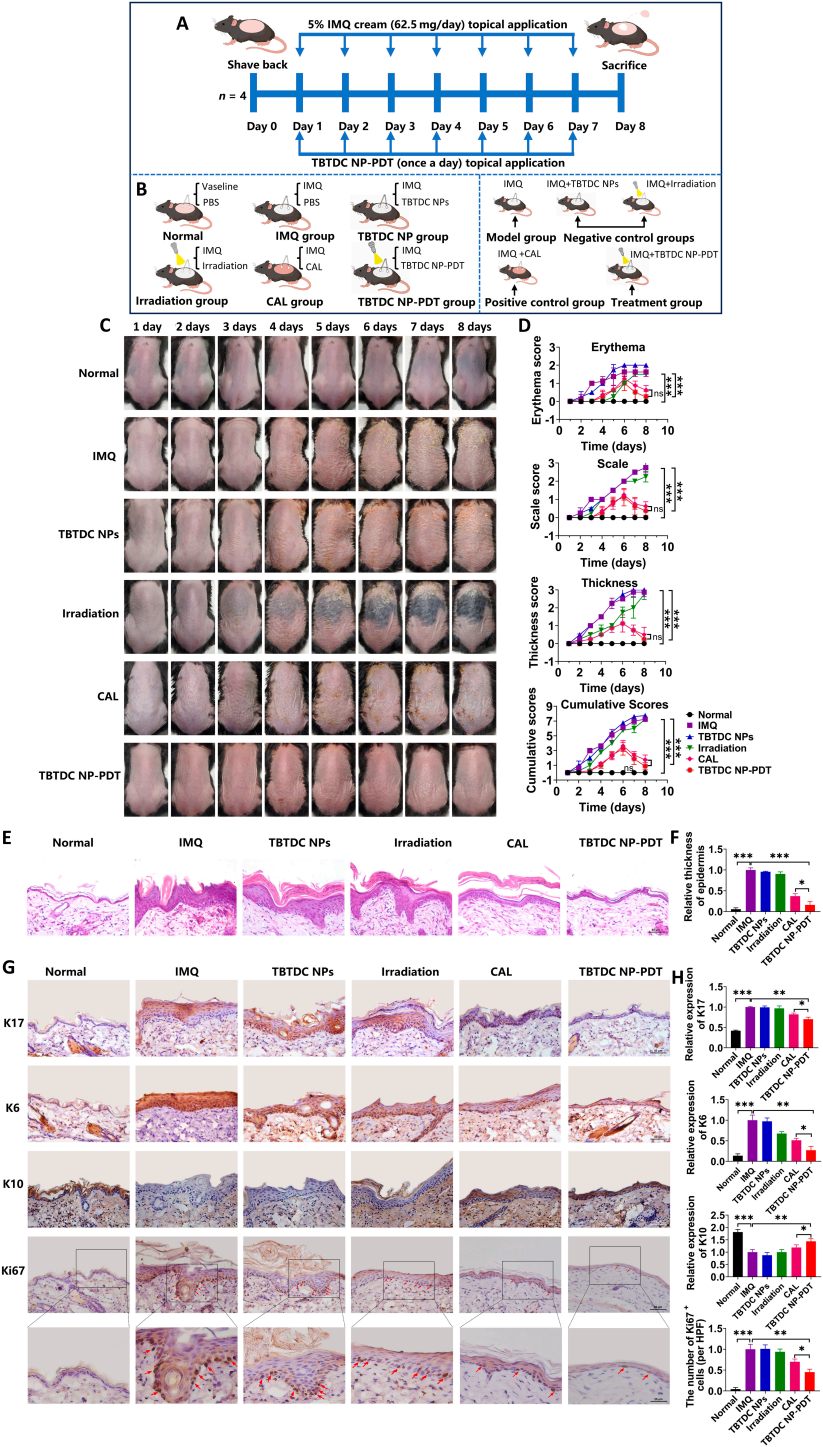
Positive effect of TBTDC NP-PDT on IMQ-induced psoriatic lesions in mice. (A and B) Diagram showing the experimental design for animal treatment. (C) Macroscopic appearance of mouse back skin from day 1 to day 8 under different treatments. (D) Erythema, scale, and thickness scores were evaluated daily based on the PASI, and statistical differences between control group and experimental group at eighth day are indicated. (E) H&E staining of skin lesions in all groups. (F) Epidermal thickness of all groups calculated by ImageJ 1.8.0. (G) IHC staining of K17, K6, K10, and Ki67 and (H) quantitative analysis of the expression of K17, K6, and K10 and number of Ki67^+^ cells in the skin lesions of all groups. Data represent the mean ± standard deviation (SD) (*n* = 4). *P < 0.05, **P < 0.01, and ***P < 0.001.

To confirm whether TBTDC NP-PDT could ameliorate skin lesions in psoriasis, the imiquimod (IMQ)-induced psoriasis-like mouse model was used in our study (Fig. S2A to D). A flowchart for this experiment is shown in Fig. 2A and B. Seven days after the application of 5% IMQ cream on the dorsal skin, IMQ-treated mice showed psoriasis-like skin lesions, such as erythema, scaling, and thickening, compared to normal mice (Fig. 2C). These symptoms were effectively ameliorated after TBTDC NP-PDT for seven consecutive days, as characterized by nearly no erythema and reduced skin scaling in IMQ-induced psoriatic mice. However, these symptoms failed to significantly improve IMQ-induced psoriasis in mice treated with TBTDC NPs or irradiation (Fig. 2C). Simultaneously, the erythema, scales, thickness, and cumulative scores in the IMQ-treated area were significantly reduced after TBTDC NP-PDT (Fig. 2D).

Furthermore, histopathological analysis showed a significant reduction in the degree of keratosis and an enrichment of the granular layer cells after TBTDC NP-PDT compared with IMQ-induced psoriatic mice (Fig. 2E). The arrangement of various cells also restored to normal levels after TBTDC NP-PDT compared with that of the IMQ-induced psoriatic mice, which were characterized by thin cuticle layers, epidermal granular layer cells, spinous layer cells, and basal columnar cell layers arranged from the outside to the inside (Fig. 2E). In addition, the epidermis was thinner after TBTDC NP-PDT than after topical CAL treatment (Fig. 2F), which has been popularly used for psoriasis [36,37]. However, these improvements were not observed in mice with IMQ-induced psoriasis treated only with TBTDC NPs or irradiation.

Keratin 17 (K17) is regarded as a specific hallmark molecule for psoriasis, and it is overexpressed in the damaged epidermis of psoriatic skin but not usually expressed in normal epidermal keratinocytes [38,39]; K6 is a marker of hyperproliferation and increases in psoriasis, while K10 is associated with normal differentiation of epidermal keratinocytes and decreases in psoriasis [38,40,41]. To assess the effect of TBTDC NP-PDT on keratinocyte proliferation and differentiation in the skin of mice with IMQ-induced psoriasis, the expression levels of K17, K6, and K10 were examined using immunohistochemistry (IHC). The results showed that the expression of K17 and K6 was significantly increased in the IMQ group but clearly decreased after TBTDC NP-PDT compared with that of the IMQ and CAL groups (Fig. 2G and H). The decreased expression of K10 in the IMQ group was significantly increased after TBTDC NP-PDT compared to that in the IMQ and CAL groups (Fig. 2G and H). Moreover, the number of Ki67^+^ cells, which are strongly present in psoriasis and correlated with the clinical severity of psoriasis [42], was also found to decrease after TBTDC NP-PDT compared to that in the IMQ and CAL groups (Fig. 2G and H). In general, TBTDC NP-PDT ameliorated the IMQ-induced psoriasis-like skin lesions in mice and exerted a therapeutic effect similar to that of topical CAL treatment.

### TBTDC NP-PDT inhibited inflammatory response in IMQ-induced psoriatic mice

**Fig. 3.**
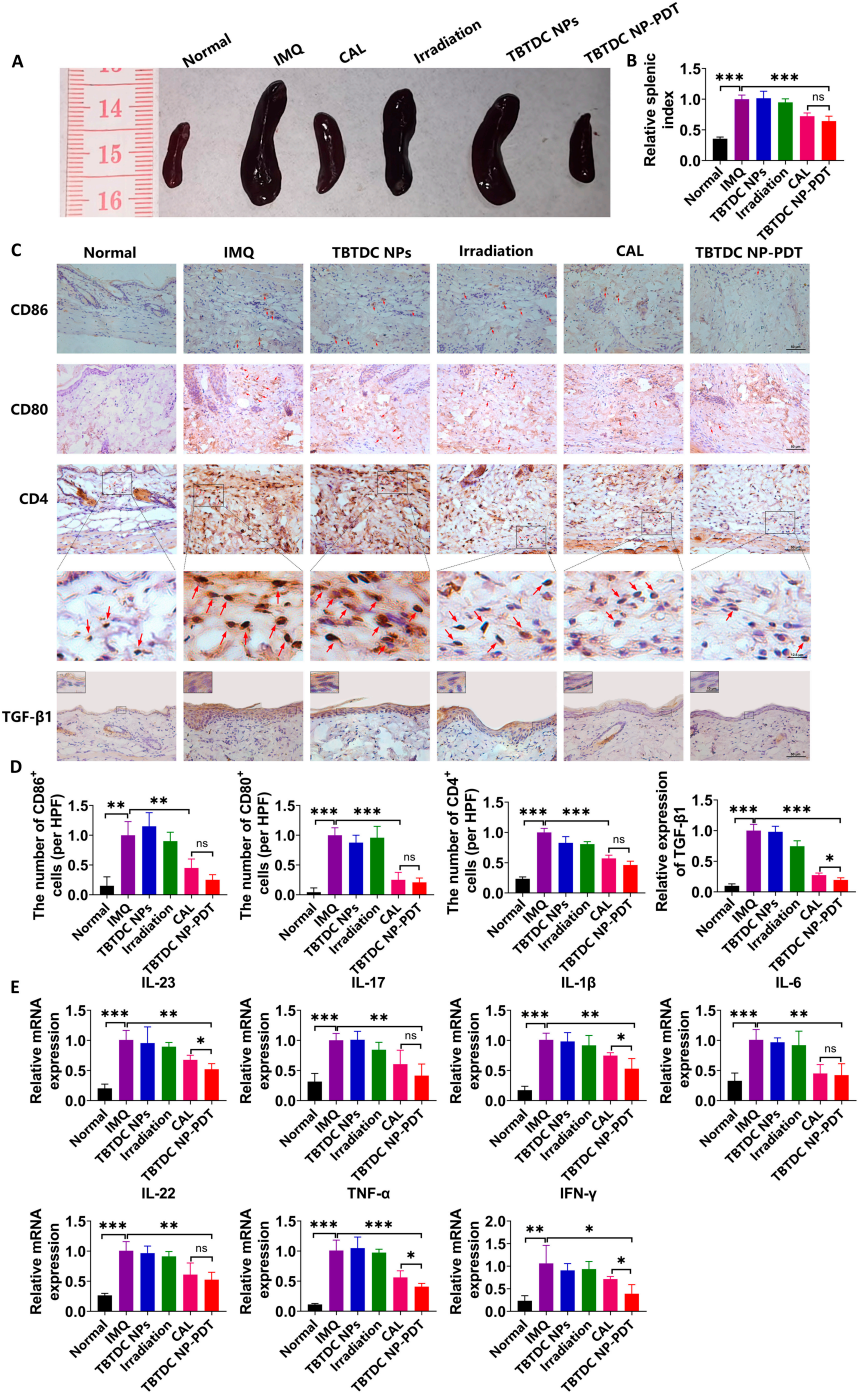
Inhibitory effects of TBTDC NP-PDT on inflammatory responses in IMQ-induced psoriatic mice. (A) Photos of mouse spleen were recorded on day 8 and quantified by the (B) spleen index (spleen weight/body weight). (C) IHC staining of CD86, CD80, CD4, and TGF-β1 and (D) quantitative analysis of the number of CD86^+^, CD80^+^, and CD4^+^ cells and expression of TGF-β1 in the skin lesions of all groups. (E) mRNA expression of psoriasis-associated inflammatory cytokines in the skin lesions of all groups. Data represent the mean ± SD (*n* = 4). *P < 0.05, **P < 0.01, and ***P < 0.001.

The inflammatory response is another important feature of psoriasis. Currently, the immune cells closely related to psoriasis are mainly dendritic cells and CD4^+^ cells. Dendritic cells produce interleukin-12 (IL-12) and IL-23, which induce naïve T cells to differentiate into CD4^+^ T cells, including T helper1 (T_H_1), T_H_17, and T_H_22 cells. CD4^+^ T cells produce tumor necrosis factor–α (TNF-α), IL-17, and IL-22, which promote keratinocyte hyperproliferation and secretion of proinflammatory chemokines, leading to greater recruitment of immune cells and ultimately amplifying and maintaining the inflammatory response [9]. To determine whether TBTDC NP-PDT could attenuate the inflammatory response in mice with IMQ-induced psoriasis, we examined the size of the spleen and the number of dendritic cells and CD4^+^ T cells in the dermis. We found that IMQ increased the relative spleen weight, while TBTDC NP-PDT significantly inhibited IMQ-induced splenomegaly (Fig. 3A and B). Mature dendritic cells express many costimulatory markers, including CD86 and CD80 (cluster of differentiation 86 and 80, respectively) [43]. In our experiment, the number of CD86^+^, CD80^+^, and CD4^+^ cells decreased after TBTDCNP-PDT compared to that in the IMQ group, as shown by IHC (Fig. 3C and D).

Transforming growth factor β1 (TGF-β1) is a cytokine crucial for T cell regulation and differentiation. Moreover, increased TGF-β1 has been found in the epidermis of psoriatic patients, and the level was closely correlated with disease severity [44,45]. IL-17, IL-23, and IL-1β are key cytokines that mediate the inflammatory response in psoriasis [46,47]. Furthermore, TNF-α, interferon-γ (IFN-γ), IL-6, and IL-22 have also been reported to be increased in psoriasis [48,49]. We further assessed whether TBTDC NP-PDT inhibited the expression of inflammatory cytokines in the skin lesions of IMQ-induced psoriatic mice by examining the expression of TGF-β1 and inflammatory cytokines by IHC and quantitative real-time reverse transcription polymerase chain reaction (qRT-PCR), respectively. In our experiment, lower TGF-β1 expression was observed after TBTDC NP-PDT, even compared with that of the CAL-positive control group (Fig. 3C and D). Furthermore, the mRNA levels of IL-23, IL17, IL-1β, IL-6, IL-22, TNF-α, and IFN-γ in lesions were highly decreased after TBTDC NP-PDT compared with that of the IMQ groups, as shown by qRT-PCR (Fig. 3E). Significantly, the mRNA levels of IL-23, IL-1β, TNF-α, and IFN-γ were considerably decreased after TBTDC NP-PDT compared with that of the CAL-positive control group, as shown by qRT-PCR (Fig. 3E). In summary, these data show that TBTDC NP-PDT inhibited the inflammatory response in mice with IMQ-induced psoriasis.

### TBTDC NP-PDT promotes keratinocytes apoptosis and autophagy in IMQ-induced psoriatic mice

**Fig. 4.**
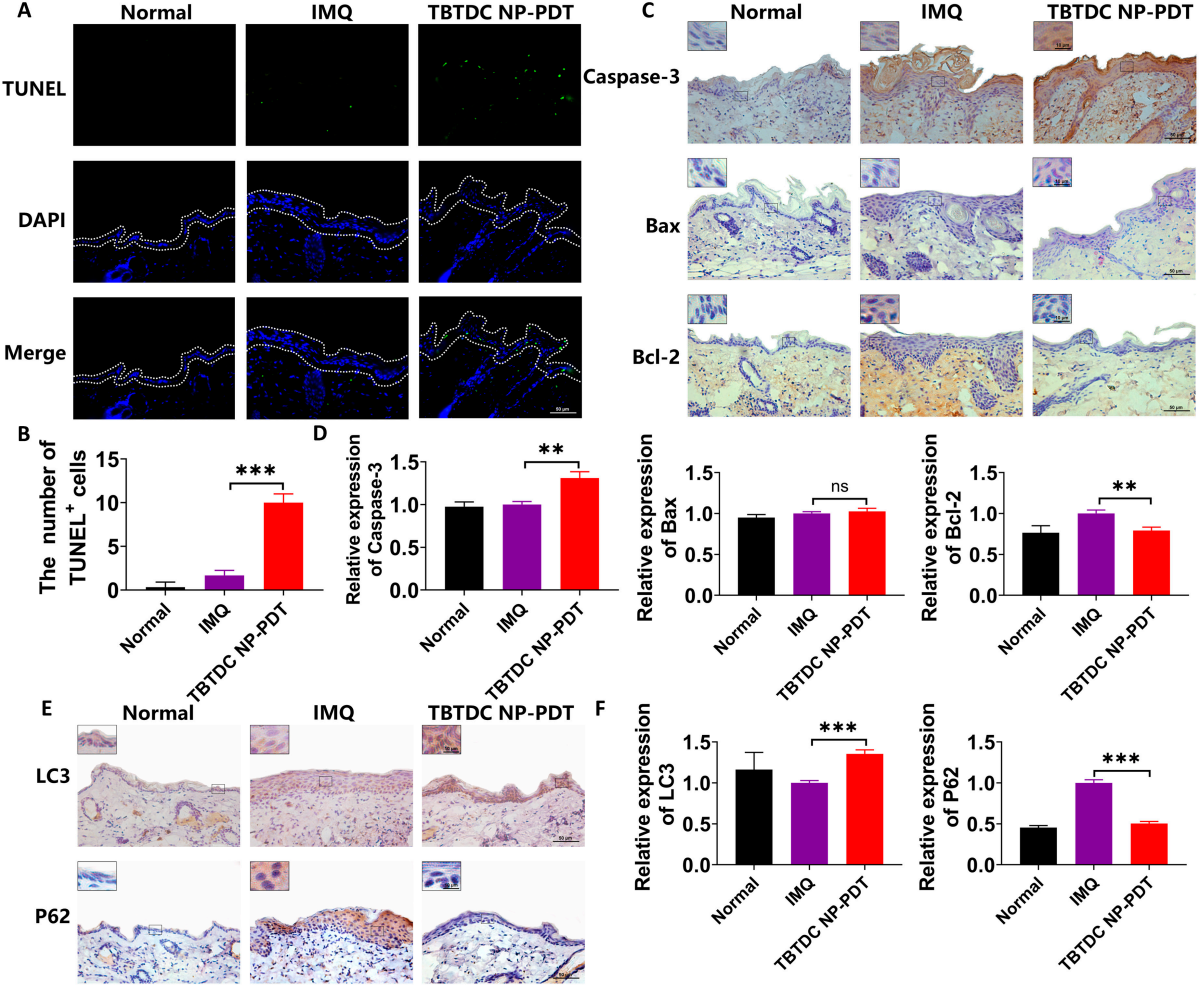
Increasing apoptosis and autophagy were found in the skin of IMQ-induced psoriatic mice after TBTDC NP-PDT. (A) TUNEL staining and (B) quantitative analysis of the number of TUNEL^+^ cells in the skin lesions of all groups. (C) IHC staining of caspase-3, Bax, and Bcl-2. (D) Quantitative analysis of the expression of caspase-3, Bax, and Bcl-2 in the skin lesions of all groups. (E) IHC staining of LC3 and P62. (F) Quantitative analysis of the expression of LC3 and P62 in the skin lesions of all groups. Data represent the mean ± SD (*n* = 3). *P < 0.05, **P < 0.01, and ***P < 0.001.

Studies have demonstrated that PDT mediates cell death by inducing apoptosis and autophagy [34,50,51]. Therefore, we examined apoptosis- and autophagy-related indicators to determine whether TBTDC NP-PDT could induce apoptosis and autophagy in psoriatic keratinocytes. In our experiment, the number of TUNEL^+^ cells was markedly decreased after TBTDC NP-PDT compared with that of the IMQ group, as shown by the terminal deoxynucleotidyl transferase-mediated deoxyuridine triphosphate nick end labeling (TUNEL) assay (Fig. 4A and B). Caspase-3, Bax, and Bcl-2 are apoptosis-related proteins, among which caspase-3 and Bax play proapoptotic roles, whereas Bcl-2 plays an anti-apoptotic role. The expression level of caspase-3 was clearly increased after TBTDC NP-PDT compared to that in the IMQ group, and the expression level of Bcl-2 was decreased; however, the expression of Bax after TBTDC NP-PDT showed no difference compared to that of the IMQ group (Fig. 4C and D). These results indicated that TBTDC NP-PDT induced keratinocyte apoptosis in mice with IMQ-induced psoriasis.

LC3 and P62 are key autophagy-related proteins. LC3 is recognized as an autophagy marker involved in autophagosome formation [52]. P62 is considered an autophagy-specific substrate that penetrates autophagosomes through interaction with LC3 and is efficiently degraded by autolysosomes during autophagy [53]. In our experiment, the expression of LC3 was increased, and the expression level of P62 was decreased after TBTDC NP-PDT compared with that in the IMQ group (Fig. 4E and F), indicating that TBTDC NP-PDT also promoted keratinocyte autophagy in IMQ-induced psoriatic mice.

### Effective intracellular uptake of TBTDC NPs in the lipopolysaccharide-induced psoriatic keratinocyte model

**Fig. 5.**
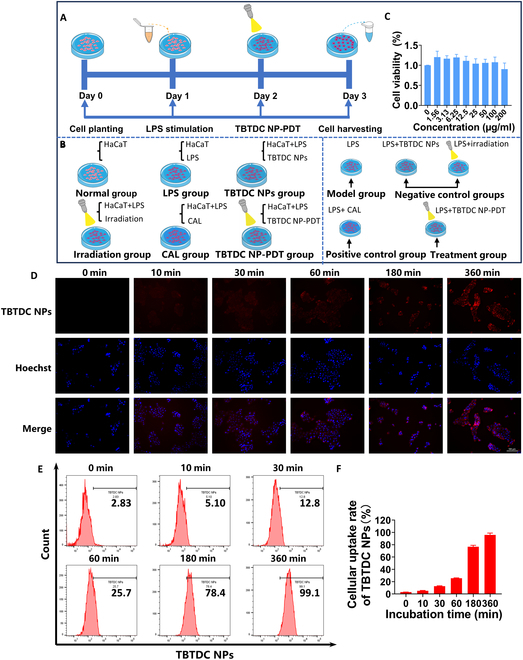
Effective and rapid intracellular uptake of TBTDC NPs observed in LPS-induced psoriatic keratinocytes. (A and B) Flowchart of cell experiments. (C) Viability of LPS-induced psoriatic keratinocytes treated with different concentrations of TBTDC NPs for 24 h using the CCK-8 method. (D) Representative fluorescence images showing the intracellular uptake of TBTDC NPs in LPS-induced psoriatic keratinocytes at various incubation time points. (E and F) Flow cytometry analysis of the intracellular uptake of TBTDC NPs in LPS-induced psoriatic keratinocytes at various times. Data represent the mean ± SD (*n* = 3).

To further explore the mechanism of TBTDC NP-PDT-mediated apoptosis and autophagy in vivo, a lipopolysaccharide (LPS)-induced psoriatic keratinocyte model was established using HaCaT cells (Fig. S4A to D). The flowchart of the cell experiment is shown in Fig. 5A and B. First, to demonstrate that TBTDC NPs are safe and nontoxic, LPS-induced psoriatic keratinocytes were treated with different concentrations of TBTDC NPs (0 to 200 μg/ml), and then a CCK-8 assay was used to measure the cell viability. No significant toxicity was observed even at concentrations as high as 200 μg/ml after incubation with TBTDC NPs for 24 h compared with that of the LPS-induced psoriatic keratinocytes group (Fig. 5C). Next, we determined the intracellular uptake and the appropriate incubation time of TBTDC NPs in LPS-induced psoriatic keratinocytes. The fluorescence microscopy photographs showed that near-infrared (NIR) fluorescence could be seen after 10 min and was clearly detected after incubation for 1 h, and the brightness gradually increased with time (Fig. 5D). Moreover, flow cytometry analysis showed that the fluorescence intensity increased with prolonged incubation (Fig. 5E and F). These results demonstrated the effective intracellular uptake of TBTDC NPs in LPS-induced psoriatic keratinocytes. The accumulation of TBTDC NPs in psoriatic keratinocytes was more than 90% after 6 h of incubation, as shown by flow cytometry (Fig. 5E and F). Thus, 6 h of incubation of TBTDC NPs was chosen for further experiments.

### TBTDC NP-PDT alleviates psoriasis-related phenotypes in LPS-induced psoriatic keratinocytes in vitro

**Fig. 6.**
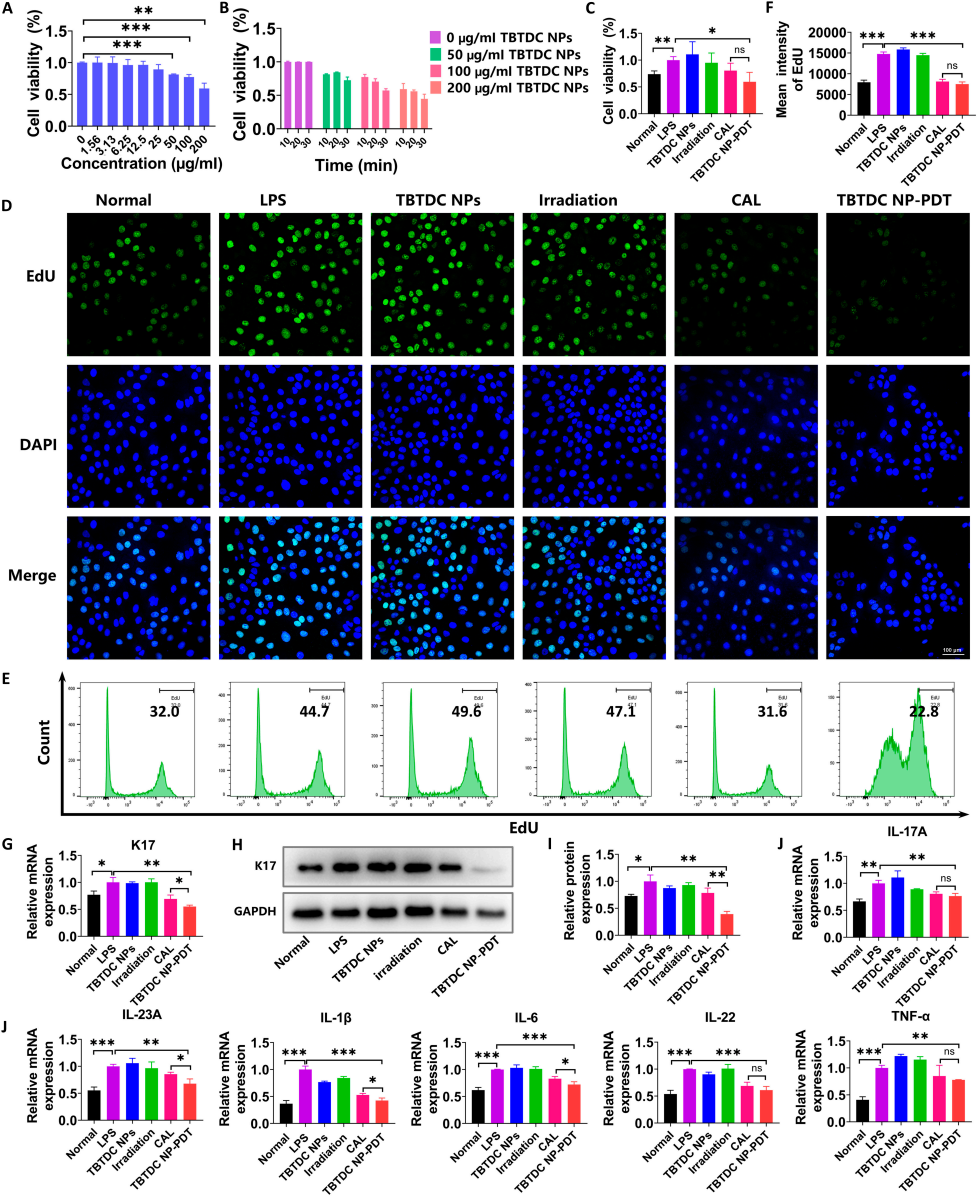
Positive effects of TBTDC NP-PDT on psoriasis-related phenotypes in LPS-induced psoriasis cell model in vitro. (A) Viability of LPS-induced psoriatic keratinocytes incubated with different concentrations of TBTDC NPs for 6 h and irradiated (520 nm, 10 mW/cm^2^) for 10 min. (B) Viability of LPS-induced psoriatic keratinocytes incubated with TBTDC NPs for the concentrations of 50, 100, and 200 μg/ml for 6 h and irradiated (520 nm, 10 mW/cm^2^) for 10, 20, and 30 min, respectively. (C) Viability of all groups under different treatments. (D) Representative fluorescence images of EdU staining in all treatment groups. (E and F) Flow cytometry analysis of EdU staining in all groups under different treatments. (G) mRNA expression of K17 in all groups under different treatments. (H) Protein levels of K17. (I) Quantitative analysis of the expression of K17 in all groups under different treatments. (J) mRNA expression of IL-17A, IL-23A, IL-1β, TNF-α, IL-6, and IL-22 in all groups under different treatments. Data represent the mean ± SD (*n* = 3). *P < 0.05, **P < 0.01, and ***P < 0.001.

To better determine the concentration of TBTDC NPs and irradiation time used for psoriatic keratinocytes in vitro, we increased the concentration of TBTDC NPs under a fixed incubation time of 6 h and irradiation time of 10 min and found that cell viability decreased with an increase in TBTDC NP concentration (Fig. 6A). TBTDC NP-PDT, especially at concentrations above 50 μg/ml, significantly decreased the viability of LPS-induced psoriatic keratinocytes (Fig. 6A). Similarly, we increased the irradiation time at doses of 50, 100, and 200 μg/ml and found that the cell viability was decreased with the increase of irradiation time (Fig. 6B). These results show that TBTDC NP-PDT significantly inhibited the viability of LPS-induced psoriatic keratinocytes in a dose- and time-dependent manner. Considering that the cell viability was decreased at a dose of 200 μg/ml compared with that of the LPS-induced psoriatic keratinocytes, a TBTDC NP concentration of 100 μg/ml and an irradiation time of 20 min were used in subsequent experiments.

As shown in Fig. 6C to F, TBTDC NP-PDT significantly inhibited cell viability and proliferation compared with that of the LPS group. Consistent with the in vivo results, we found that TBTDC NP-PDT significantly reduced K17 expression at the mRNA and protein levels compared to the LPS group and CAL-positive control group, as indicated by qRT-PCR and Western blot (WB) in vitro (Fig. 6G to I). Furthermore, we examined a set of inflammatory cytokines, including IL-17A, IL-23A, IL-1β, TNF-α, IL-6, and IL-22, at the mRNA level in LPS-induced psoriatic keratinocytes, which are closely related to psoriasis in vivo. The results showed that IL-17A, IL-23A, IL-1β, TNF-α, IL-6, and IL-22 were clearly decreased at mRNA levels after TBTDC NP-PDT compared with that of the LPS group, and the expression of IL-23A, IL-1β, and IL-6 was significantly reduced even compared with that of the CAL-positive control group (Fig. 6J). Overall, these results confirm that TBTDC NP-PDT alleviates psoriasis-related phenotypes to some extent in LPS-induced psoriatic keratinocytes in vitro.

### TBTDC NP-PDT induced ROS production and promoted apoptosis in LPS-induced psoriatic keratinocytes

**Fig. 7.**
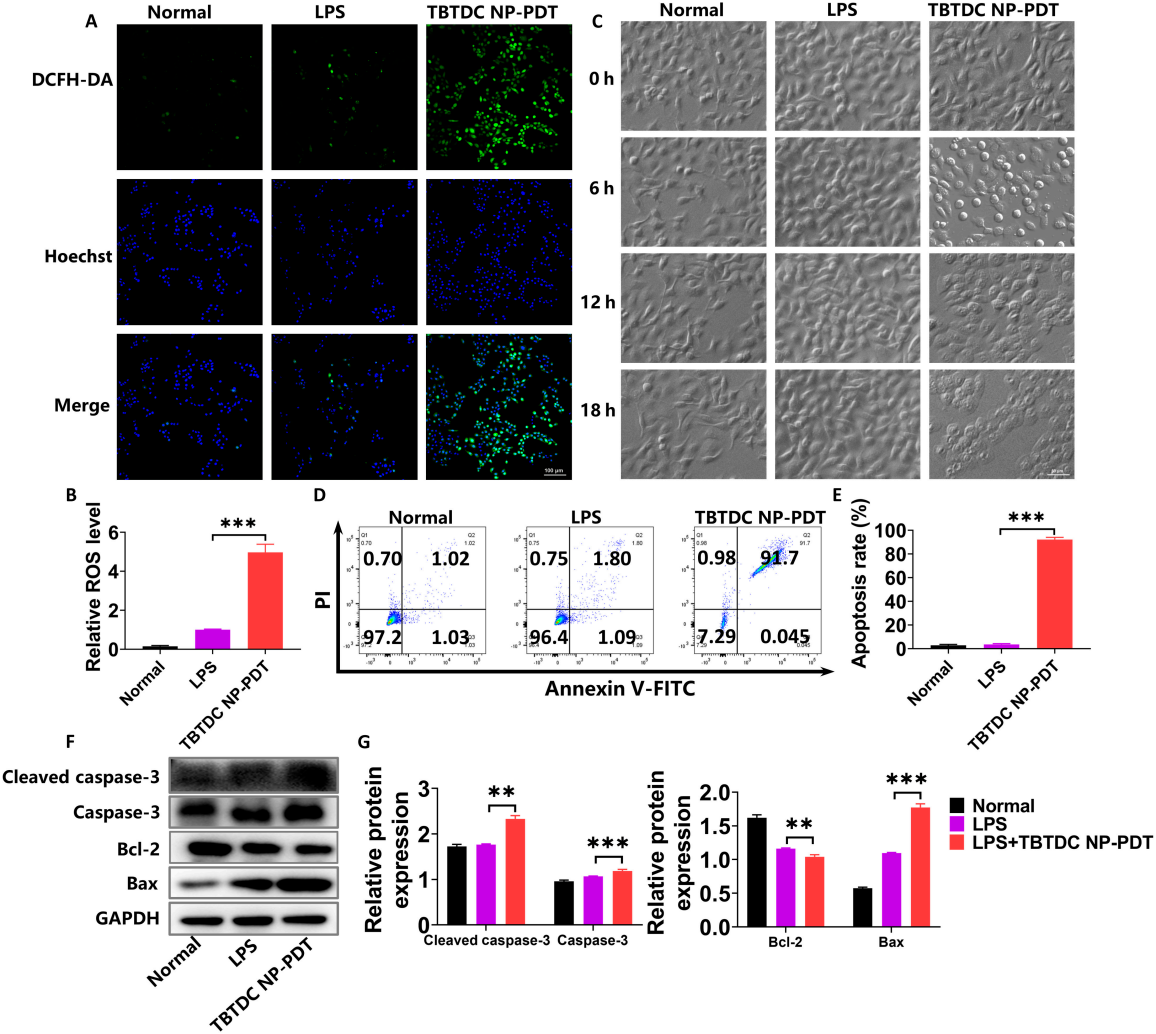
Excessive ROS and apoptosis detected in LPS-induced psoriatic keratinocytes after TBTDC NP-PDT. (A) Confocal images of ROS generation in LPS-induced psoriatic keratinocytes after TBTDC NP-PDT. (B) Levels of ROS production in LPS-induced psoriatic keratinocytes after TBTDC NP-PDT analyzed by fluorescent microplate reader. (C) Representative morphology images of LPS-induced psoriasis keratinocytes recorded after TBTDC NP-PDT for different times. (D and E) Cell apoptosis rate of LPS-induced psoriatic keratinocytes after TBTDC NP-PDT analyzed by flow cytometry. (F) Protein levels of caspase-3, cleaved caspase-3, Bax, and Bcl-2. (G) Quantitative analysis of the expression of caspase-3, cleaved caspase-3, Bax, and Bcl-2 in LPS-induced psoriatic keratinocytes after TBTDC NP-PDT. Data represent the mean ± SD (*n* = 3). *P < 0.05, **P < 0.01, and ***P < 0.001.

Because TBTDC NPs are highly efficient organic photosensitizers with AIE and have a high ROS production rate, 2′,7′-dichlorofluorescein diacetate (DCFH-DA) was employed as an ROS indicator to explore ROS production after TBTDC NP-PDT in LPS-induced psoriatic keratinocytes. The generation of intracellular ROS was obviously increased after TBTDC NP-PDT compared to that in the LPS group, as shown by fluorescence microscopy and fluorescent microplate reader analysis (Fig. 7A and B). To determine the mechanism of increased apoptosis caused by TBTDC NP-PDT in LPS-induced psoriatic keratinocytes, we first observed the morphology of the cells after TBTDC NP-PDT under a microscope. We found that the morphology of LPS-induced psoriatic keratinocytes changed from a spindle shape to a round shape and finally separated from the bottom of the dish over time after TBTDC NP-PDT (Fig. 7C). Next, the apoptotic rate was determined and analyzed using annexin V-fluorescein isothiocyanate (FITC)/propidium iodide (PI) double staining and flow cytometry. Similar to the in vivo experiments, significantly increased numbers of apoptotic cells were found after TBTDC NP-PDT compared to that in the LPS group (Fig. 7D and E). Importantly, WB analysis also demonstrated that TBTDC NP-PDT up-regulated the expression of caspase-3, cleaved caspase-3 (active form of caspase-3), and Bax, while it down-regulated the expression of Bcl-2 in LPS-induced psoriatic keratinocytes compared with that of the LPS group (Fig. 7F and G). These results showed that TBTDC NP-PDT induced ROS generation and promoted apoptosis in LPS-induced psoriatic keratinocytes.

### TBTDC NP-PDT alleviated psoriasis-like phenotype and promoted apoptosis through the massive generation of ROS in vitro

**Fig. 8.**
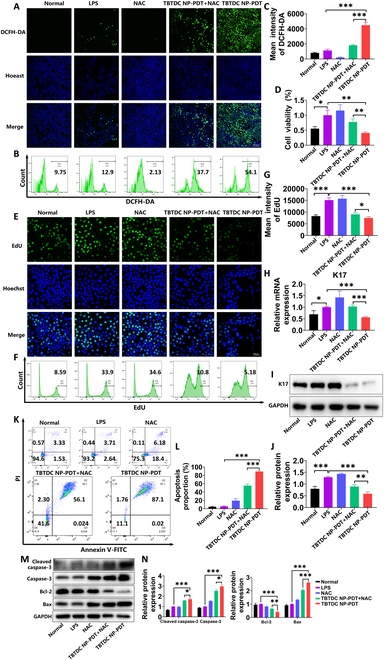
Alleviation of the psoriasis-like phenotype and promotion of apoptosis in LPS-induced psoriatic keratinocytes after TBTDC NP-PDT via ROS. (A) Confocal images of ROS generation in LPS-induced psoriatic keratinocytes after TBTDC NP-PDT with or without NAC. (B and C) Levels of ROS production in LPS-induced psoriatic keratinocytes after TBTDC NP-PDT with or without NAC analyzed by flow cytometry. (D) Viability of LPS-induced psoriatic keratinocytes after TBTDC NP-PDT with or without NAC. (E) Representative fluorescence images of EdU staining of LPS-induced psoriatic keratinocytes after TBTDC NP-PDT with or without NAC. (F and G) Flow cytometry analysis of EdU staining of LPS-induced psoriatic keratinocytes after TBTDC NP-PDT with or without NAC. (H) mRNA expression of K17 of LPS-induced psoriatic keratinocytes after TBTDC NP-PDT with or without NAC. (I) Protein levels of K17. (J) Quantitative analysis of the expression of K17 in LPS-induced psoriatic keratinocytes after TBTDC NP-PDT with or without NAC. (K and L) Cell apoptosis rate of LPS-induced psoriatic keratinocytes after TBTDC NP-PDT with or without NAC analyzed by flow cytometry. (M) Protein levels of caspase-3, cleaved caspase-3, Bax, and Bcl-2. (N) Quantitative analysis of the expression of caspase-3, cleaved caspase-3, Bax, and Bcl-2 in LPS-induced psoriatic keratinocytes after TBTDC NP-PDT with or without NAC. Data represent the mean ± SD (*n* = 3). *P < 0.05, **P < 0.01, and ***P < 0.001.

High ROS production induced by PDT is crucial in cell damage [30,31]. To further investigate whether the apoptosis-promoting effect of TBTDC NP-PDT on LPS-induced psoriatic keratinocytes is mediated by ROS, *N*-acetyl-cysteine (NAC), a ROS scavenger, was used. The generation of ROS induced by TBTDC NP-PDT was significantly decreased after the addition of NAC (1 mM) (Fig. 8A to C). We further found that the inhibition of cell viability and cell proliferation resulting from TBTDC NP-PDT in LPS-induced psoriatic keratinocytes was greatly rescued by NAC, as detected by the CCK-8 and 5-ethynyl-2’-deoxyuridine (EdU) assays (Fig. 8D to G). This finding confirmed to some extent the vital role of ROS produced by TBTDC NP-PDT in mediating the inhibited growth of LPS-induced psoriatic keratinocytes. Furthermore, the decreased expression of K17 induced by TBTDC NP-PDT was rescued by NAC, as detected by qRT-PCR and WB analysis (Fig. 8H to J). Therefore, TBTDC NP-PDT-mediated alleviation of the psoriasis phenotype in LPS-induced psoriatic keratinocytes depends on ROS production.

Next, we examined whether TBTDC NP-PDT-mediated apoptosis in LPS-induced psoriatic keratinocytes occurred via the production of large amounts of ROS. The number of apoptotic cells induced by TBTDC NP-PDT was significantly decreased after the addition of NAC, as detected and analyzed by annexin V-FITC/PI double staining and flow cytometry (Fig. 8K and L). At the same time, up-regulation of caspase-3, cleaved caspase-3, and Bax and down-regulation of Bcl-2 resulting from TBTDC NP-PDT in LPS-induced psoriasis keratinocytes were significantly rescued by NAC, as detected by WB analysis (Fig. 8M and N). These findings showed that TBTDC NP-PDT-mediated apoptosis occurred via the production of ROS. In summary, these results suggest that TBTDC NP-PDT alleviates the psoriasis-like phenotype and promotes apoptosis by efficiently generating ROS in vitro.

### TBTDC NPs target the endoplasmic reticulum in LPS-induced psoriatic keratinocytes, which promotes autophagy by ERS after TBTDC NP-PDT

**Fig. 9.**
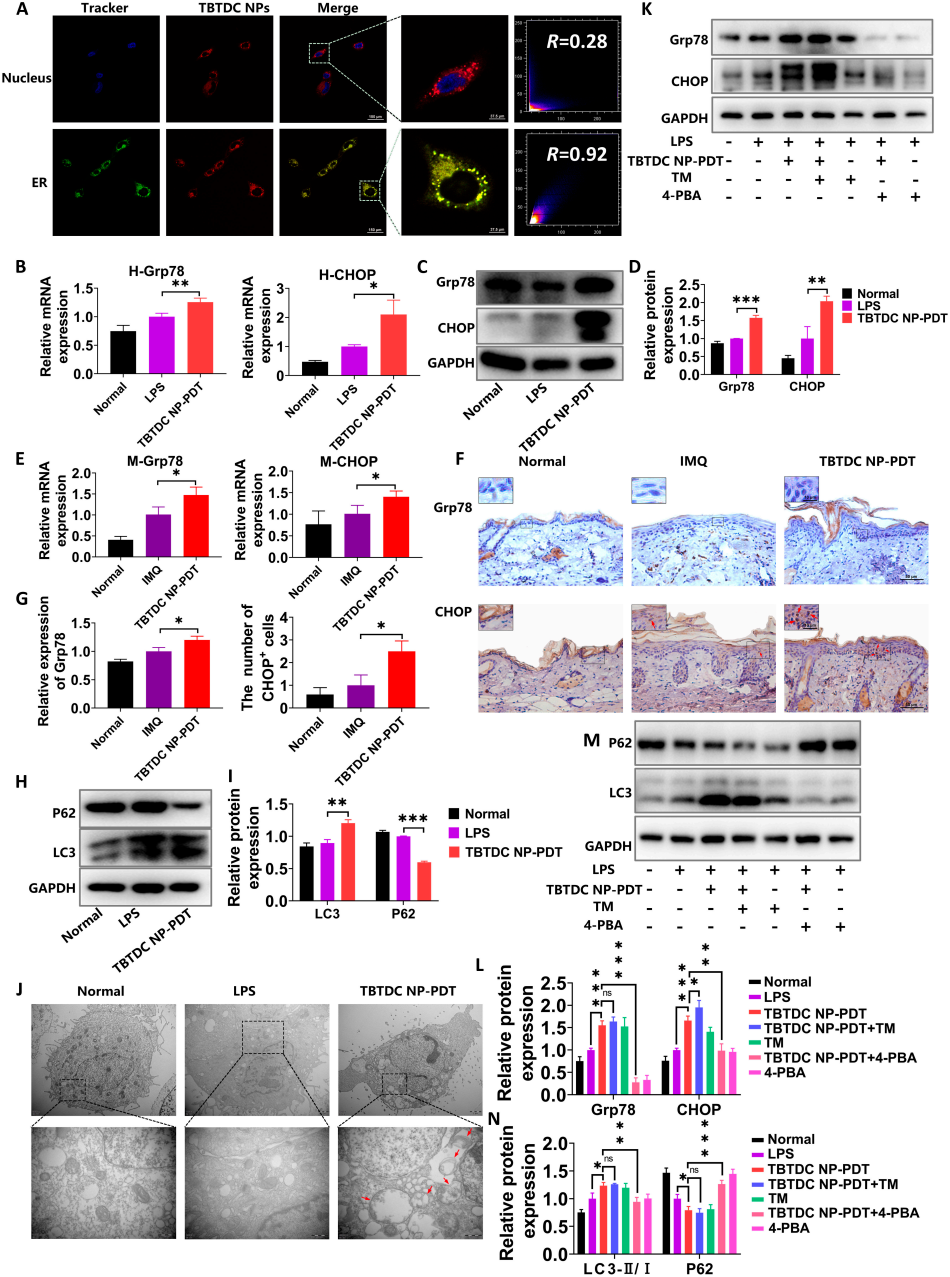
TBTDC NPs target endoplasmic reticulum in LPS-induced psoriatic keratinocytes, leading to the induction of ERS after TBTDC NP-PDT, promoting autophagy. (A) CLSM images of LPS-induced psoriatic keratinocytes incubated with TBTDC NPs and then costained with Hoechst 33258 and ER-Tracker Green. (B) mRNA expression of Grp78 and CHOP in LPS-induced psoriatic keratinocytes after TBTDC NP-PDT. (C) Protein levels of Grp78 and CHOP and (D) quantitative analysis of the expression of Grp78 and CHOP in LPS-induced psoriatic keratinocytes after TBTDC NP-PDT. (E) mRNA expression of Grp78 and CHOP in IMQ-induced psoriatic mice after TBTDC NP-PDT. (F) IHC staining of Grp78 and CHOP and (G) quantitative analysis of the expression of Grp78 and the number of CHOP^+^ cells in IMQ-induced psoriatic mice after TBTDC NP-PDT. (H) Protein levels of LC3 and P62. (I) Quantitative analysis of the expression of LC3 and P62 in LPS-induced psoriatic keratinocytes after TBTDC NP-PDT. (J) Autophagosome formation observed by TEM in LPS-induced psoriatic keratinocytes after TBTDC NP-PDT. (K) Protein levels of Grp78 and CHOP. (L) Quantitative analysis of the expression of Grp78 and the number of CHOP^+^ cells in LPS-induced psoriatic keratinocytes after TBTDC NP-PDT with or without the addition of TM and 4-PBA. (M) Protein levels of LC3 and P62. (N) Quantitative analysis of the expression of LC3 and P62 in LPS-induced psoriatic keratinocytes after TBTDC NP-PDT with or without the addition of TM and 4-PBA. Data represent the mean ± SD (*n* = 3). *P < 0.05, **P < 0.01, and ***P < 0.001.

Specific localization of photosensitizers to certain organelles can result in a targeted attack that causes greater trauma to target cells and eventually maximizes PDT [29]. To determine the organelle-targeting specificity of the TBTDC NPs in LPS-induced psoriatic keratinocytes, a colocalization experiment was performed. As presented in Fig. 9A, Pearson’s correlation coefficients for the nucleus and endoplasmic reticulum were 0.28 and 0.92, respectively, and the NIR fluorescence of TBTDC NPs coincided with the green fluorescence of ER-Tracker Green. These results demonstrated that TBTDC NPs specifically targeted the endoplasmic reticulum in LPS-induced psoriatic keratinocytes. Previous studies have shown that irradiation-induced activation of endoplasmic reticulum-localizing photosensitizers causes oxidative damage to the endoplasmic reticulum, leading to the induction of ERS [51], which can be marked by glucose-regulated protein 78 (Grp78) and CCAAT enhancer binding protein homologous protein (CHOP) [54,55]. Therefore, we assessed whether TBTDC NP-PDT induces ERS in LPS-induced psoriatic keratinocytes. The expression of Grp78 and CHOP was clearly increased in LPS-induced psoriatic keratinocytes after TBTDC NP-PDT compared to that in the LPS group, as detected by qRT-PCR and WB assays, respectively (Fig. 9B to D). Interestingly, increased expression of Grp78 and CHOP was also observed in the skin of IMQ-induced psoriatic mice after TBTDC NP-PDT compared to that in the IMQ group, as detected by qRT-PCR and IHC assays (Fig. 9E to G). These findings indicated that TBTDC NPs specifically targeted the endoplasmic reticulum and caused ERS in psoriatic keratinocytes in vitro and in vivo.

Studies have shown that ERS is an important mechanism that induces autophagy [56,57]. During autophagy, cytoplasmic LC3 (LC3-I) enzymatically cleaves a small polypeptide fragment and converts it to the membrane type (LC3-II). Thus, the ratio of LC3-II/LC3-I can be used to evaluate the level of autophagy [58,59]. To determine whether keratinocyte autophagy observed in TBTDC NP-PDT is mediated by ERS, we first detected autophagy-related molecules in LPS-induced psoriatic keratinocytes by WB in vitro. Consistent with the in vivo experiments, a significantly enhanced ratio of LC3-II/LC3-I and reduced expression of P62 were observed in LPS-induced psoriatic keratinocytes after TBTDC NP-PDT compared to those in the LPS group (Fig. 9H and I). Furthermore, a clear increase in autophagosomes was found after TBTDC NP-PDT compared with that of the LPS group, as detected by transmission electron microscopy (TEM) (Fig. 9J). To further evaluate the effect of ERS on autophagy, tunicamycin (TM), a mixture of homologous nucleoside antibiotics that cause the accumulation of unfolded proteins in the endoplasmic reticulum, was used to activate ERS [60], and 4-phenylbutyric acid (4-PBA), a chemical chaperone, was used to inhibit ERS [61]. The results showed that the increased levels of CHOP and Grp78 increased by TBTDC NP-PDT in LPS-induced psoriatic keratinocytes were further increased by TM (1 μg/ml) but significantly suppressed by 4-PBA (1 mM) (Fig. 9K and L), which indicated that ERS was efficiently activated by TM and inhibited by 4-PBA. Furthermore, we found that the enhanced ratio of LC3-II/LC3-I induced by TBTDC NP-PDT was further increased by TM, while the reduction of P62 was decreased by TM (Fig. 9M and N). Moreover, both the enhanced ratio of LC3-II/LC3-I and the reduction in P62 induced by TBTDC NP-PDT were rescued by 4-PBA (Fig. 9M and N). Collectively, these results confirmed that TBTDC NPs targeted the endoplasmic reticulum in LPS-induced psoriatic keratinocytes, resulting in the induction of ERS after TBTDC NP-PDT and eventually promoting autophagy.

### Increasing autophagy resulting from TBTDC NP-PDT also promoted apoptosis in LPS-induced psoriatic keratinocytes

**Fig. 10.**
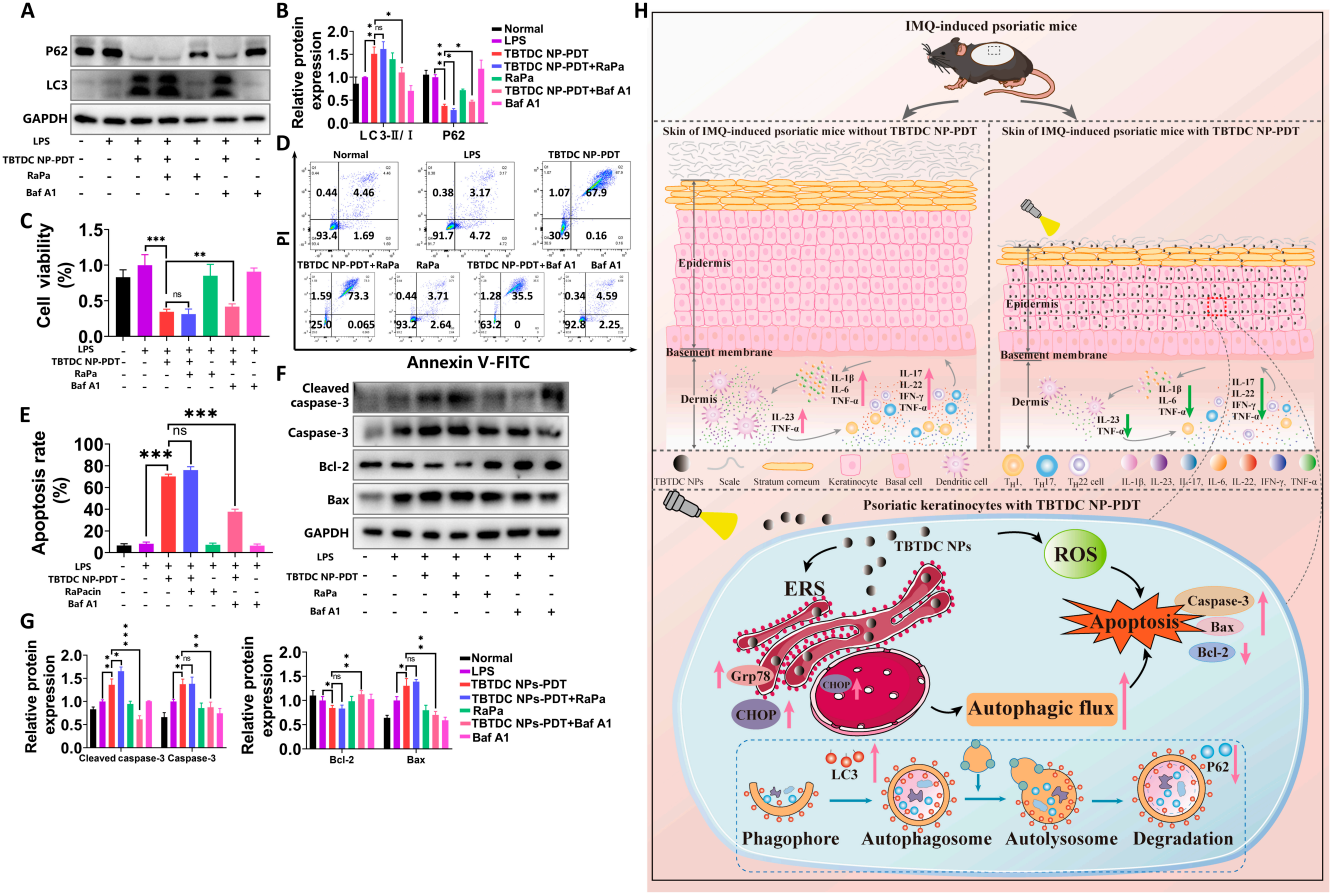
Increased autophagy induced by TBTDC NP-PDT also promoted apoptosis in LPS-induced psoriatic keratinocytes. (A) Protein levels of LC3 and P62 and (B) quantitative analysis of the expression of LC3 and P62 in LPS-induced psoriatic keratinocytes after TBTDC NP-PDT with or without the addition of RaPa and Baf A1. (C) Viability of LPS-induced psoriatic keratinocytes after TBTDC NP-PDT with or without the addition of RaPa and Baf A1. (D and E) Cell apoptosis rate of LPS-induced psoriatic keratinocytes after TBTDC NP-PDT with or without the addition of RaPa and Baf A1, as analyzed by flow cytometry. (F) Protein levels of caspase-3, cleaved caspase-3, Bax, and Bcl-2. (G) Quantitative analysis of the expression of caspase-3, cleaved caspase-3, Bax, and Bcl-2 in LPS-induced psoriatic keratinocytes after TBTDC NP-PDT with or without the addition of RaPa and Baf A1. (H) Schematic view illustrating the therapeutic role and regulatory mechanisms of TBTDC NP-PDT in psoriasis. Data represent the mean ± SD (*n* = 3). *P < 0.05, **P < 0.01, and ***P < 0.001.

Recent studies have shown that autophagy, which promotes cell survival, may inhibit apoptosis, while excessive autophagy may promote apoptosis [34,35]. To determine whether autophagy induced by TBTDC NP-PDT in LPS-induced psoriatic keratinocytes could promote apoptosis, rapamycin (RaPa), a potent and specific mammalian target of rapamycin (mTOR) inhibitor, was used to activate autophagy [62]. In contrast, bafilomycin A1 (Baf A1), a late-stage autophagy inhibitor, was used to block autophagosome and lysosome fusion [63]. RaPa (0.1 μM) effectively increased the enhanced ratio of LC3-II/LC3-I and decreased the reduction of P62 induced by TBTDC NP-PDT, while Baf A1 (10 nM) markedly increased the down-regulation of P62 induced by TBTDC NP-PDT (Fig. 10A and B), which indicated that autophagy was efficiently activated by RaPa and inhibited by Baf A1. In addition, the reduced cell viability of LPS-induced psoriatic keratinocytes resulting from TBTDC NP-PDT was significantly increased by Baf A1 (Fig. 10C) but reduced by RaPa, as shown by the CCK-8 assay (Fig. 10C). This finding indicates the positive role of autophagy in the inhibition of cell growth. Next, we used an annexin V-FITC/PI apoptosis detection kit combined with flow cytometry to analyze the apoptosis rate. We found that the increased apoptosis rate induced by TBTDC NP-PDT in LPS-induced psoriatic keratinocytes was further increased by RaPa but reduced by Baf A1 (Fig. 10D and E). Moreover, the increased expression of apoptosis-related molecules induced by TBTDC NP-PDT, including caspase-3, cleaved caspase-3, and Bax, was enhanced by RaPa but reduced by Baf A1, while the decreased expression of Bcl-2 induced by TBTDC NP-PDT was reduced by RaPa but enhanced by Baf A1, as shown by WB (Fig. 10F and G). These results demonstrated that increased autophagy from TBTDC NP-PDT also promoted apoptosis in LPS-induced psoriatic keratinocytes. The schematic graph depicting the mechanism of TBTDC NP-PDT alleviating psoriasis was displayed in Fig. 10H, which enlightened that TBTDC NP-PDT was an effective candidate for psoriasis.

## Discussion

Psoriasis is one of the most common skin diseases and usually causes great inconvenience to patients and seriously damages their physical and mental health [6,7]. Therefore, it is necessary to develop efficient therapeutic strategies to relieve this burden on patients. In this study, we used AIE-specific photosensitizer TBTDC NP-mediated PDT as a psoriasis treatment. At the same time, CAL, a commonly used physical therapy drug, was employed as a positive control to evaluate the therapeutic effect of TBTDC NP-PDT on psoriasis in vivo and in vitro, and further explore the mechanism.

As a highly efficient multifunctional organic photosensitizer with AIE function, TBTDC NPs showed higher ROS production than commercial Ce6 and RB, suggesting an excellent PDT effect of TBTDC NPs. Moreover, TBTDC NPs showed no toxicity to HaCaT cells or psoriatic keratinocytes and were effectively taken up by psoriatic keratinocytes, indicating their good safety and biocompatibility. Our group has previously demonstrated that TBTDC NP-PDT can effectively inhibit tumor growth [28]. These characteristics indicate that TBTDC NPs may be an ideal photosensitizer, which have an extremely high application value for the treatment of skin diseases.

IMQ is a Toll-like receptor 7/8 ligand agonist that induces psoriasis-like dermatitis in mice, closely resembling human psoriasis [64]. IMQ-treated mice showed symptoms of psoriasis-like lesions such as erythema, scales, epidermal hyperplasia, splenomegaly, and inflammatory cell infiltration, which are also closely linked to the increasing expression of K17 [38] and K6, and the decreasing expression of K10 [40,41]. Surprisingly, the TBTDC NP-PDT significantly ameliorated these symptoms. In our study, TBTDC NP-PDT significantly ameliorated the psoriasis-like symptoms and decreased the expression of K17 and K6 but increased the expression of K10 in the IMQ-induced psoriatic mice. We also evaluated the effects of TBTDC NP-PDT in the psoriasis cell model. In our study, LPS (0.1 μg/ml) stimulation of HaCaT cells for 24 h (LPS-induced psoriatic keratinocytes) [65] increased cell viability, proliferation, and K17 expression. However, TBTDC NP-PDT alleviated LPS-induced hyperproliferation and K17 overexpression. Thus, our results suggest that TBTDC NP-PDT ameliorates the psoriasis-like phenotype both in vivo and in vitro.

Furthermore, we found that TBTDC NP-PDT significantly inhibited IMQ-induced splenomegaly. The number of dendritic cells, marked by CD86 and CD80 antibodies and playing a critical role in the local inflammatory response in psoriasis [66], and CD4^+^ cells in the dermis was also decreased after TBTDC NP-PDT. Last but not least, TBTDC NP-PDT attenuated IMQ-induced up-regulation of inflammatory cytokines, including TGF-β1, IL-1β, IL-17, IL-23, TNF-α, IFN-γ, IL-6, and IL-22, which are important proinflammatory cytokines involved in psoriasis pathogenesis in the skin lesions of mice. Meanwhile, LPS-induced up-regulation of inflammatory cytokines, including IL-1β, IL-17A, IL-23A, TNF-α, IL-6, and IL-22, was also down-regulated in psoriatic keratinocytes after TBTDC NP-PDT in vitro. Our study demonstrated that TBTDC NP-PDT inhibits inflammatory responses in vivo and reduces the release of inflammatory cytokines from psoriatic keratinocytes in vitro.

In terms of mechanism, we found that TBTDC NP-PDT significantly enhanced the apoptosis of keratinocyte in vivo, including increased TUNEL^+^ cells, up-regulated caspase-3, and down-regulated Bcl-2 (an anti-apoptotic protein), which was further confirmed in vitro by increasing the expression of caspase-3, cleaved caspase-3, and Bax while decreasing Bcl-2. Moreover, it also significantly up-regulated LC3 and down-regulated P62 in vitro and in vitro. In addition, a clear increase in LC3-II/LC3-I ratio and autophagosomes was observed after TBTDC NP-PDT in vitro. These results strongly indicate that TBTDC NP-PDT can enhance apoptosis and autophagy in vivo and in vitro.

Previous studies have shown that PDT generally promotes apoptosis by producing ROS [32,33]. In our experiment, TBTDC NPs showed an extremely high ^1^O_2_ yield upon light irradiation compared to Ce6 and RB. Furthermore, intracellular ROS production was significantly increased in LPS-induced psoriatic keratinocytes after TBTDC NP-PDT. Finally, the ROS scavenger NAC significantly decreased the high apoptosis rate and the expression of caspase-3, cleaved caspase-3, and Bax caused by TBTDC NP-PDT, but up-regulated Bcl-2, suggesting that apoptosis induced by TBTDC NP-PDT was dependent on the generation of ROS. In addition, the inhibition of cell viability, cell proliferation, and K17 expression resulting from TBTDC NP-PDT in LPS-induced psoriatic keratinocytes were also rescued by NAC. Thus, these results revealed that TBTDC NP-PDT alleviated the psoriasis-like phenotype and promoted apoptosis through the efficient generation of ROS.

During PDT, photosensitizers with organellar targeting function are more effective in damaging cells [29]. TBTDC NPs were found to specifically target the endoplasmic reticulum, with Pearson’s correlation coefficients of 0.92 in psoriatic keratinocytes by organelle colocalization experiments. However, the reason why TBTDC NPs have such high endoplasmic reticulum-targeting activity in psoriatic keratinocytes is unclear. Interestingly, TBTDC NP-PDT obviously up-regulated the levels of the ERS-related proteins Grp78 and CHOP in psoriatic keratinocytes in vitro and in vivo. ERS is an important mechanism underlying autophagy induction [56,57]. In our study, inhibiting ERS by 4-PBA reduced autophagy induced by TBTDC NP-PDT, while activating ERS by TM increased autophagy, strongly suggesting that autophagy induced by TBTDC NP-PDT is dependent on ERS. Recently, several studies reported that autophagy promotes or inhibits apoptosis [34,35]. In this study, TBTDC NP-PDT-induced apoptosis was significantly reduced by the autophagy inhibitor Baf A1 and increased by the autophagy activator RaPa, revealing that increased autophagy resulting from TBTDC NP-PDT could further promote apoptosis in LPS-induced psoriatic keratinocytes. This study indicates that TBTDC NPs are safe and effective photosensitizing agents against psoriasis. However, a few limitations were observed in our experiment. For example, the mechanism by which TBTDC NP-PDT regulates immune cells and inflammatory cytokines associated with psoriasis and the process by which TBTDC NPs target the endoplasmic reticulum remains to be investigated.

In summary, this study reports for the first time that TBTDC NPs with AIE characteristics can mediate PDT to cure psoriasis-like phenotypes through apoptosis induced directly by ROS and indirectly by ERS-induced autophagy in vivo and in vitro. Furthermore, this study will contribute to the development of innovative and highly efficient photosensitizers for psoriasis treatment.

## Materials and Methods

### Preparation, measurements, and characterization of TBTDC NPs

TBTDC NPs were fabricated from TBTDC (molecular formula: C_36_H_22_N_6_S_3_; molecular weight: 634.80 Da) as described in our previous study [28].

A Bruker AVANCE III 400 spectrometer was used to record ^1^H nuclear magnetic resonance (NMR) spectra at 400 MHz using CDCl_3_ as the solvent. The reference for ^1^H shifts was CDCl_3_ at 7.26 ppm. Ultraviolet–visible (UV–vis) absorption spectra were monitored using a UV–vis spectrophotometer (Shimadzu UV-2600i, Japan). Fluorescence spectra were measured using a Transient Steady-state Fluorescence Spectrometer (Edinburgh, FLS 980). The dynamic light scattering size distribution of the TBTDC NPs was examined using a Malvern Zetasizer Nano instrument. TEM images of TBTDC NPs were obtained using a JEM 2100 microscope operated at an acceleration of 200 kV.

To prepare the suspension for the animal experiments, TBTDC NPs at a concentration of 200 μg/ml were dispersed in phosphate-buffered solution (PBS) and then sonicated in an ice bath for 25 min prior to use.

To prepare stock solutions for the cell experiments, TBTDC NPs at 2 mg/ml concentration were evenly dispersed in PBS, sterilized with a 0.22-μm filter, and then stored at 4 °C in the dark. The working solution was diluted in HaCaT cell medium and dispersed by ultrasound in an ice bath for 15 min before use.

### Evaluation of singlet oxygen generation

9,10-Anthracenediyl-bis(methylene)-dimalonic acid (ABDA; Shanghai Macklin Biochemical Co. Ltd.) was used to detect the ^1^O_2_ generation ability of TBTDC NPs. A 2-ml TBTDC NP (5 μM) aqueous solution containing 10 μl of ABDA (50 μM) was irradiated by white light (400 to 700 nm, 50 mW/cm^2^) for 0 to 10 min at intervals of 30 s to measure the UV–vis spectra. Commercial standardized photosensitizers, including Ce6 and RB, were purchased from J&K Scientific Ltd. and Shanghai Macklin Biochemical Co. Ltd., respectively, and used for comparison analyses.

### Experimental animals

Male, specific pathogen-free (SPF) C57BL/6J mice (8 to 10 weeks) were purchased from Southern Medical University Laboratory Animal Center (Guangzhou, China) and reared in an SPF environment with fixed irradiation (12:12 h light–dark cycle), constant temperature (22 ± 1 °C), and humidity (60% to 80%), and all animals were allowed free access to food and water. All mice were caged and acclimated to the new environment for 5 days before the start of the experiment. All animal experiments in this study were approved by the Southern Medical University Experimental Animal Ethics Committee (No. L2018133).

### Psoriasis mouse model and treatments

IMQ cream (Mingxin Pharmaceuticals, Sichuan, China) was used to generate a mouse model of psoriasis according to previously reported protocols [65,67,68]. Briefly, the back hairs of the mice were shaved to fully expose the skin under anesthesia (80 mg/kg pentobarbital sodium, intraperitoneal injection). A dose of 62.5 mg of 5% IMQ cream was applied to the hairless area once daily in the morning for seven consecutive days.

Mice were randomly divided into six groups (*n* = 4 per group). For the normal group, an appropriate amount of Vaseline was applied daily to the hair-free areas of the back. For the IMQ group (model group), mice did not receive any treatment except IMQ induction. For the TBTDC NP group (negative control group 1), the IMQ-treated areas were covered with TBTDC NPs (3 mg/kg), and black plastic sheeting was applied over the TBTDC NP-covered area to prevent exposure to irradiation. For the irradiation group (negative control group 2), the IMQ-treated areas were covered with PBS and then irradiated with a high-power light-emitting diode (LED) light (10 mW/cm^2^, 520 nm) for 10 min daily. For the CAL (vitamin D3 analog) group (positive control group), CAL ointment (Huabang Pharmaceuticals, Chongqing, China) was topically applied to the IMQ-treated areas daily. For the TBTDC NP-PDT group (treatment group), the IMQ-treated areas were covered by TBTDC NPs for 4 h and then irradiated using the high-power LED light (10 mW/cm^2^, 520 nm) for 10 min. Mice were sacrificed on day 8, and fresh skin was harvested for fixation or freezing. A schematic diagram describing the design of the animal experiment is shown in Fig. 2A and B.

### Evaluation of pathological indexes

Dorsal skin appearance was recorded daily using a camera starting from the first day of treatment, and the degree of psoriasis-like lesions was dynamically monitored and scored using a modified scoring system based on the clinical psoriasis area severity index (PASI). The erythema, scale, and thickness scores ranged from 0 to 4 points: 0, none; 1, slight; 2, moderate; 3, marked; and 4, very marked. The sum of the erythema, scale, and thickness scores was the cumulative score.

### H&E staining

Hematoxylin and eosin (H&E) staining was performed for histological examination as previously described [69]. Briefly, the dorsal skin of IMQ-treated mice was fixed in 4% paraformaldehyde (PFA) (pH 7.2) for 48 h. After dehydration treatment, the specimens were embedded in paraffin, and then 4-μm-thick sections were rehydrated and stained with H&E. Images were obtained using a biological microscope (DM40008, Leica, Germany), and epidermal thickness was calculated using ImageJ 1.8.0.

### IHC assay

For the IHC analysis, the rehydrated sections were successively placed in citrate buffer (pH 6.0) at 95 °C for 10 min and then incubated with 3% H_2_O_2_ at 37 °C for 15 min, blocking solution containing 10% goat serum at room temperature for 1 h, and primary antibodies overnight at 4 °C. The antibodies included anti-Ki67 (1:200, Abcam, UK); anti-K10 and anti-K6 (1:500 and 1:500, respectively, all BioLegend, CA, USA); anti-CD4 (1:300, Immunoway, China); anti-CD86 (1:100, ABclonal, USA); anti-K17, anti-CD80, anti-TGF-β1, anti-LC3, anti-P62, anti-Bax, anti-Bcl-2, and anti-caspase-3 (1:250, 1:300, 1:200, 1:200, 1:500, 1:2,000, 1:400, and 1:200, respectively, Proteintech, USA); and anti-Grp78 and anti-CHOP (1:200 and 1:50, respectively, GeneTex, SC, USA). After washing three times with PBS, the sections were incubated with horseradish peroxidase (HRP)-labeled goat anti-rabbit immunoglobulin G (IgG) or anti-mouse IgG (all ZSGB-BIO, Beijing, China) at 37 °C for 1 h. After washing again with PBS, immunoreactivity was detected by incubation with diaminobenzidine (DAB) stain (Solarbio, Beijing, China). Images were obtained using a biological microscope (DM40008; Leica, Germany). Quantitative analysis of related indicators was performed using ImageJ 1.8.0.

### qRT-PCR analysis

FastPure Cell/Tissue Total RNA Isolation Kit V2 (Vazyme, Nanjing, China) was used to extract total RNA from mouse skin tissue or HaCaT keratinocytes according to the manufacturer’s instructions. The absorbance values at 260 and 280 nm were measured using a spectrophotometer (Molecular Devices, San Jose, CA, USA) to determine the concentration and purity of total RNA. The mRNA samples were reverse-transcribed to complementary DNA by ReverTra Ace qRT-PCR RTMaster Mix (TOYOBO, Japan).

qRT-PCR was performed using SYBR Green Real-time PCR Master Mix (TOYOBO, Japan) in CFX96 Touch Real-Time PCR Detection System (Bio-Rad, USA). The qRT-PCR procedure consisted of initial denaturation at 95 °C for 30 s, followed by 40 cycles of denaturation at 95 °C for 5 s, annealing at 55 °C for 20 s, and extension at 72 °C for 15 s. Glyceraldehyde-3-phosphate dehydrogenase (GAPDH) and β-actin were used as internal references. The relative mRNA expression levels were analyzed using the 2^−ΔΔCt^ method [70]. Primer sequences targeting genes in mice and humans were shown in Tables S1 and S2.

### TUNEL staining

TUNEL detection solution (Beyotime, China) was dropped onto the sections and incubated at 37 °C in the dark for 60 min according to the manufacturer’s instructions.

### Cell culture

HaCaT keratinocytes were purchased from Cyagen Biotechnology Co. Ltd. (Guangzhou, China) and grown in Dulbecco’s modified Eagle’s medium (Gibco, Suzhou, China) containing 10% fetal bovine serum (Biological Industries, Israel) and 1% penicillin/streptomycin (Gibco, USA) at 37 °C in a humidified atmosphere of 5% CO_2_. The culture medium was changed every other day. When the cells reached 80% confluence, they were harvested using trypsin (Gibco, USA) and plated in well plates.

### Psoriasis cell model and treatments

HaCaT keratinocytes (2 × 10^5^ cells per well) were cultured for 12 h in advance, the culture medium was replaced, and the cells were treated with LPS (0.1 μg/ml, Sigma-Aldrich, USA) for 24 h to construct the psoriasis cell model [65,67].

The grouping of cell experiments was consistent with that in the animal experiments. Briefly, cells were divided into six groups and subjected to different treatments. In the normal group (model group), cell suspensions were added to well plates without LPS induction. In the LPS group, cells did not receive any treatment except for LPS induction. For the TBTDC NP group (negative control group 1), LPS-induced psoriatic keratinocytes were incubated with 100 μg/ml TBTDC NPs without irradiation. In the irradiation group (negative control group 2), LPS-induced psoriatic keratinocytes were irradiated with a high-power LED light (520 nm, 10 mW/cm^2^) for 20 min without TBTDC NPs. For the TBTDC NP-PDT group (treatment group), LPS-induced psoriatic keratinocytes were incubated with 100 μg/ml TBTDC NPs in the dark for 6 h and then irradiated by the high-power LED light (520 nm, 10 mW/cm^2^) for 20 min and finally incubated for additional 18 h. For the CAL group (positive control group), 0.5 μM CAL was added to LPS-induced psoriatic keratinocytes. A schematic diagram describing the design of the cell experiment is shown in Fig. 5A and B.

### Detection of intracellular TBTDC NP uptake

To measure the intracellular uptake of TBTDC NPs, HaCaT keratinocytes were plated in six-well plates (5 × 10^5^ cells per well) 12 h in advance and then treated with LPS (0.1 μg/ml) for 24 h. The culture medium was discarded after 24 h, and cells were incubated with TBTDC NPs (10 μg/ml) in the dark for the indicated periods. The cells were collected at different incubation time points (0, 10, 30, 60, 180, and 360 min) and analyzed using flow cytometry (LSRFortessa X-20, BD, USA) and fluorescence microscopy (Olympus, Japan). The mean fluorescence intensity of TBTDC NPs in the cells was recorded under uniform testing conditions.

### Cell viability assay

Cell viability was estimated using the CCK-8 (Beyotime, China) assay. HaCaT keratinocytes were plated in 96-well plates (6 × 10^3^ cells per well) and treated with LPS (0.1 μg/ml) for 24 h. To detect the safety of TBTDC NPs, LPS-induced psoriasis keratinocytes were incubated with fresh medium containing various concentrations (0, 1.56, 3.13, 6.25, 12.5, 25, 50, 100, and 200 μg/ml) of TBTDC NPs in darkness for 24 h. After the cells were washed twice using PBS, 10 μl of CCK-8 solution was added to each well, and the cells were incubated for another 2 h. The optical density (OD) was recorded at 450 nm using a microplate reader.

To select the appropriate incubation concentration, TBTDC NPs with the same concentration gradient were incubated with LPS-induced psoriatic keratinocytes for 6 h and then exposed to an energy density of 10 mW/cm^2^ using the high-power LED light (wavelength 520 nm) for 10 min and finally incubated for additional 18 h. Similarly, to select the appropriate irradiation time, TBTDC NPs with concentrations of 50, 100, and 200 μg/ml were incubated with LPS-induced psoriatic keratinocytes for 6 h exposed to the high-power LED light (520 nm, 10 mW/cm^2^) for 10, 20, and 30 min, respectively, and finally incubated for additional 18 h. The following CCK-8 assays were performed in the same way as described above. Cell viability of the different treatments was evaluated using the aforementioned method.

### EdU assay

For the EdU assay (Beyotime, China), cells (2 × 10^5^ cells per well) were plated in 12-well plates. After TBTDC NP-PDT, the cells were incubated with EdU working solution for 2 h, fixed with 4% PFA, and permeabilized with 0.3% Triton X-100 PBS. Then, a sequence of reaction solutions was added to the wells, and the nuclei were stained with Hoechst. Cell proliferation was then visualized using a fluorescence microscope and detected using flow cytometry.

### WB analysis

HaCaT cells were plated in six-well plates (5 × 10^5^ cells per well). After TBTDC NP-PDT, RIPA Lysis Buffer (ECOTOP SCIENTIFIC, ES-8148, 100 ml), protease inhibitor cocktail (Bimake, Houston, Texas, USA, B14001), and phosphatase inhibitor cocktail (Bimake, Houston, Texas, USA, B15001) were used to lyse the cells cultured in six-well plates for 20 min on ice. Cell lysates were centrifuged for 15 min at 14,462*g* and then heated in 5× sodium dodecyl sulfate–polyacrylamide gel electrophoresis (SDS-PAGE) Loading Buffer (Genstar, China) at 100 °C for 10 min. The protein concentration of the harvested cell supernatant was determined using a BCA kit (Thermo Fisher Scientific, MA, USA, 23227). Approximately 20 μg of protein was separated using SDS-PAGE and then transferred to polyvinyl difluoride membranes (Merck Millipore, Darmstadt, Germany) and blocked with QuickBlock Blocking Buffer (Beyotime, China, P0252) for 1 h. The membranes were incubated with primary antibodies overnight at 4 °C, including anti-K17, anti-caspase-3, anti-LC3, anti-P62, and anti-GAPDH (1:2,000, 1:1,000, 1:1,000, 1:5,000, and 1:10,000, respectively, Proteintech, USA), anti-Bax (1:500, Cell Signaling Technology, USA), anti-Bcl-2 (1:500, Santa Cruz Biotechnology, USA), and anti-Grp78 and anti-CHOP (1:5,000 and 1:2,000, respectively, GeneTex, SC, USA). Tris-buffered saline containing Tween 20 (TBST) was used to wash the membranes three times for 10 min each, followed by incubation with HRP-conjugated secondary antibodies (1:10,000, Proteintech, USA) for 1 h at room temperature. After three washes with TBST, an enhanced chemiluminescence ECL kit (P10200, NCM Biotech, Suzhou, China) was used to detect proteins in an automated chemiluminescence image analysis system (Tanon, Shanghai, China). The expression levels of proteins were quantified using ImageJ 1.8.0.

### Measurement of intracellular ROS production

Briefly, the culture medium was discarded, and cells were washed twice with PBS after TBTDC NP-PDT, followed by incubation with basal culture medium (without serum) containing 10 μM DCFH-DA probes (Nanjing Jiancheng Bioengineering Institute) for 1 h in the dark. Then, the fluorescence intensity was observed using a fluorescence microscope and detected by a fluorescence microplate reader or flow cytometry.

For the ROS inhibition experiments, cells were preincubated with 1 mM ROS scavenger NAC for 1 h before TBTDC NP-PDT and washed twice with PBS after TBTDC NP-PDT. The production of intracellular ROS was then detected using the aforementioned method.

### Annexin V-FITC/PI analysis

After PDT, the cells were cultured for an additional 18 h and stained with FITC-conjugated annexin V and PI (Beyotime) following the manufacturer’s guidelines. Flow cytometry was used to analyze the final cell suspensions after staining.

### Organelle colocalization experiments

HaCaT keratinocytes (5 × 10^4^ cells per well) were plated in a confocal dish for 12 h and then subjected to LPS induction. After LPS-induced psoriatic keratinocytes were incubated with TBTDC NPs (10 μg/ml) for 6 h, the culture medium was changed, and the cells were washed three times with PBS. The cells were then costained with the ER-Tracker Green (Beyotime, China) for 15 min and Hoechst 33258 (Beyotime, China) for 5 min. The culture medium was finally discarded, and the cells were washed twice with PBS. The NIR fluorescence of the TBTDC NPs, green fluorescence of ER-Tracker Green, and blue fluorescence of Hoechst were determined via confocal laser scanning microscopy (CLSM). Pearson’s correlation coefficients were analyzed using ImageJ 1.8.0.

### TEM analysis

After performing TBTDC NP-PDT for 18 h, the cells were harvested and fixed with 2.5% glutaraldehyde for 1 h at room temperature, before being transferred to 4 °C overnight. The fixed cells were treated with 1% buffered osmium tetroxide for 1 h at 4 °C, followed by sequential dehydration with a graded ethanol series. The cell samples were then fixed and embedded, sectioned into thin sections, and finally stained with 3% uranyl acetate/lead citrate. Subsequently, images were obtained using an H-7650 TEM (Hitachi, Japan).

### Statistical analysis

All data were presented as the mean ± SD of at least three biological replicates. One-way analysis of variance and unpaired Student’s *t* tests were utilized for statistical analyses using GraphPad Prism 8.0. A value of *P* < 0.05 was considered statistically significant.

## Data Availability

The datasets used and/or analyzed in the current study are available from the corresponding author on reasonable request.
