# Supplementary material for: Aggregation-Induced Emission-Active Photosensitizer-Mediated Photodynamic Therapy for Anti-Psoriasis
Source: Research (Wash D C). 2024 Jun 6;7:0344. doi: 10.34133/research.0344 (PMC11301704; doi:10.34133/research.0344)
Supplement: Supplementary 1 — Supplementary_Materials (Figs. S1 to S6) Tables S1 and S2 [file research.0344.f1.docx]

***Supplementary Materials***

**Aggregation-induced emission-active photosensitizer-mediated photodynamic therapy for anti-psoriasis**

Ping Zhu^1,2,3^**^†^**, Zhaoji Wu^4^**^†^**, Zhilu Yang^5^, Tingting Tang^1,2,3^, Yunhui Liao^4^, Wen Zhao^6^, Ying Huang^7^, Tao Chen^8^, Junjie Li^9^, Chunmei Nong^10^, Zhenzhen Wu^10^, Guodong Hu^10*^, Yanshan Liu^4*^, Yinghua Chen^2,3*^

^1^Department of Histology and Embryology, NMPA Key Laboratory for Safety Evaluation of Cosmetics, School of Basic Medical Sciences, Southern Medical University, Guangzhou, 510515, China

^2^Dongguan People’s Hospital Biobank, Affiliated Dongguan Hospital, Southern Medical University, Dongguan, Guangdong 523059, China

^3^Guangdong Provincial Key Laboratory of Construction and Detection in Tissue Engineering, School of Basic Medical Sciences, Southern Medical University, Guangzhou, 510515, China

^4^NMPA Key Laboratory for Research and Evaluation of Drug Metabolism & Guangdong Provincial Key Laboratory of New Drug Screening, School of Pharmaceutical Sciences, Southern Medical University, Guangzhou 510515, China

^5^Dongguan Key Laboratory of Smart Biomaterials and Regenerative Medicine, The Tenth Affiliated Hospital, Southern Medical University, Dongguan, Guangdong, 523059, China

^6^Department of Medical Imaging, Guangzhou Women and Children's Medical Center, National Children's Medical Center for South Central Region, Guangzhou 510515, China

^7^Health management center, The Tenth Affiliated Hospital, Southern Medical University, Dongguan, Guangdong 523059, China

^8^Nanfang Hospital Biobank, Clinical Research Center, Nanfang Hospital, Southern Medical University, No. 1838 Guangzhou Avenue, Guangzhou 510515, China.

^9^Department of Dermatology, The Tenth Affiliated Hospital, Southern Medical University, Dongguan, Guangdong 523059, China

^10^Department of Respiratory and Critical Care Medicine, Dongguan Key Laboratory of Clinical Translation of Basic Research on Respiratory diseases, The Tenth Affiliated Hospital, Southern Medical University, Dongguan, Guangdong 523059, China

^*^Address correspondence to: Guodong Hu ([huguodong@smu.edu.cn](mailto:huguodong@smu.edu.cn)); Yanshan Liu ([liuys9@smu.edu.cn](mailto:liuys9@smu.edu.cn)); Yinghua Chen ([mrchch@126.com](mailto:mrchch@126.com))

**^†^**These authors contributed equally to this work.

**
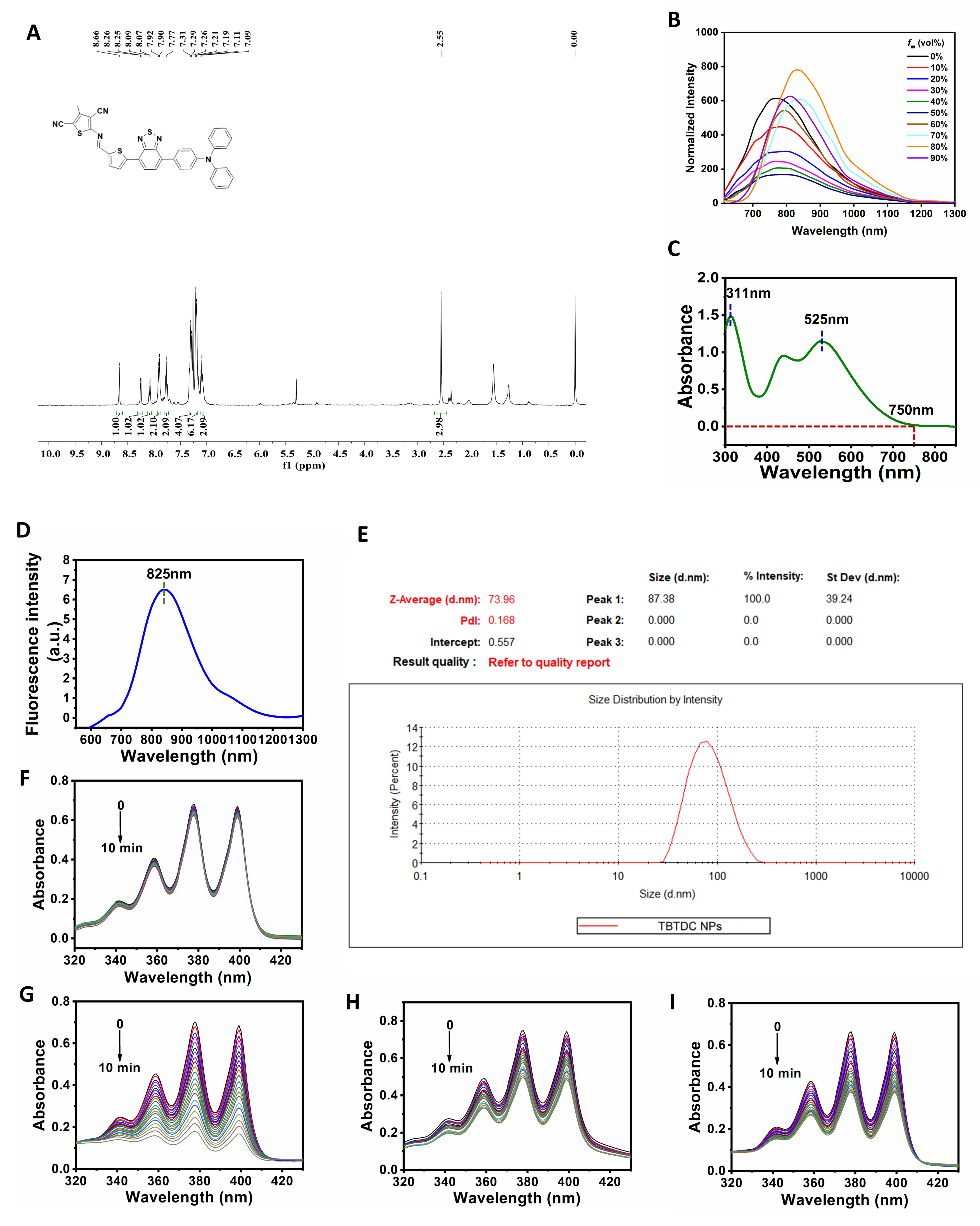
**

**Figure. S1. Characterization of TBTDC NPs. (A)** ^1^H NMR spectrum of TBTDC in chloroform-d; **(B)** Fluorescence spectra of TBTDC (10 μM) in THF/water mixtures with different *f*_w_s (*λ*_ex_ = 520 nm); **(C)** Absorption and **(D)** emission (λex = 560 nm) spectra of TBTDC NPs in THF solution; **(E)** Hydrodynamic diameter of the TBTDC NPs was measured using DLS; **(F)** Degradation of ABDA and **(G)** ABDA with TBTDC NPs, **(H)** Ce6 and **(I)** RB under light irradiation (400–700 nm, 50 mW/cm^2^), [TBTDC NPs] = 5 μM, [Ce6] = 5 mΜ, [RB] = 5 μM, [ABDA] = 50 μM.


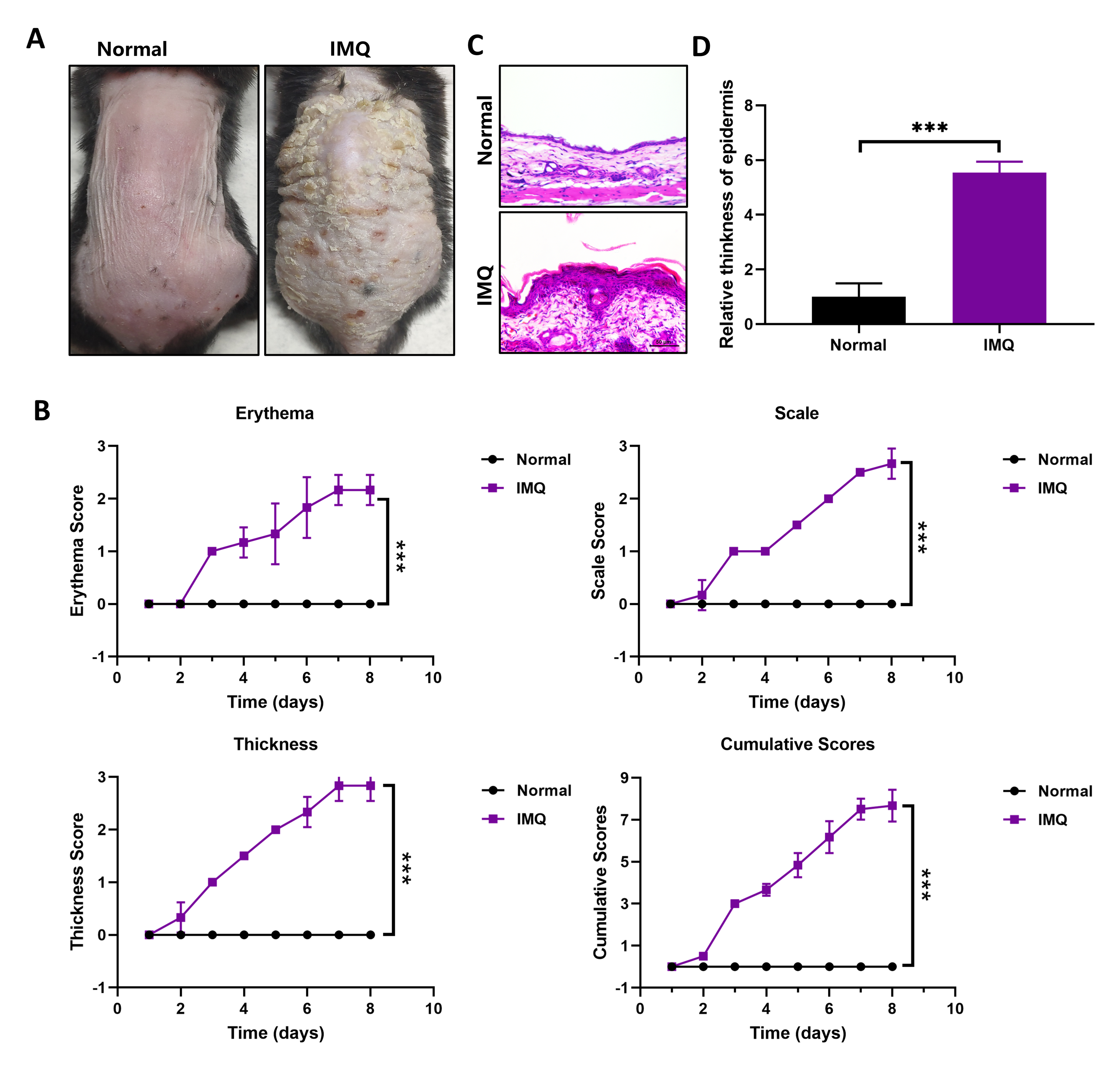


**Figure. S2. Construction of a psoriasis mouse model in vitro. (A)** Macroscopic appearance of normal and IMQ-induced mice after seven days of continuous application of IMQ; **(B)** Erythema, scale, and thickness scores were evaluated daily based on the PASI, and the statistical difference between normal and IMQ-induced mice on the 8th day is indicated; **(C)** H&E staining of back skin of normal and IMQ-induced mice. Bar, 50 μm; **(D)** Epidermal thickness of normal and IMQ-induced mice was calculated using ImageJ 1.8.0.


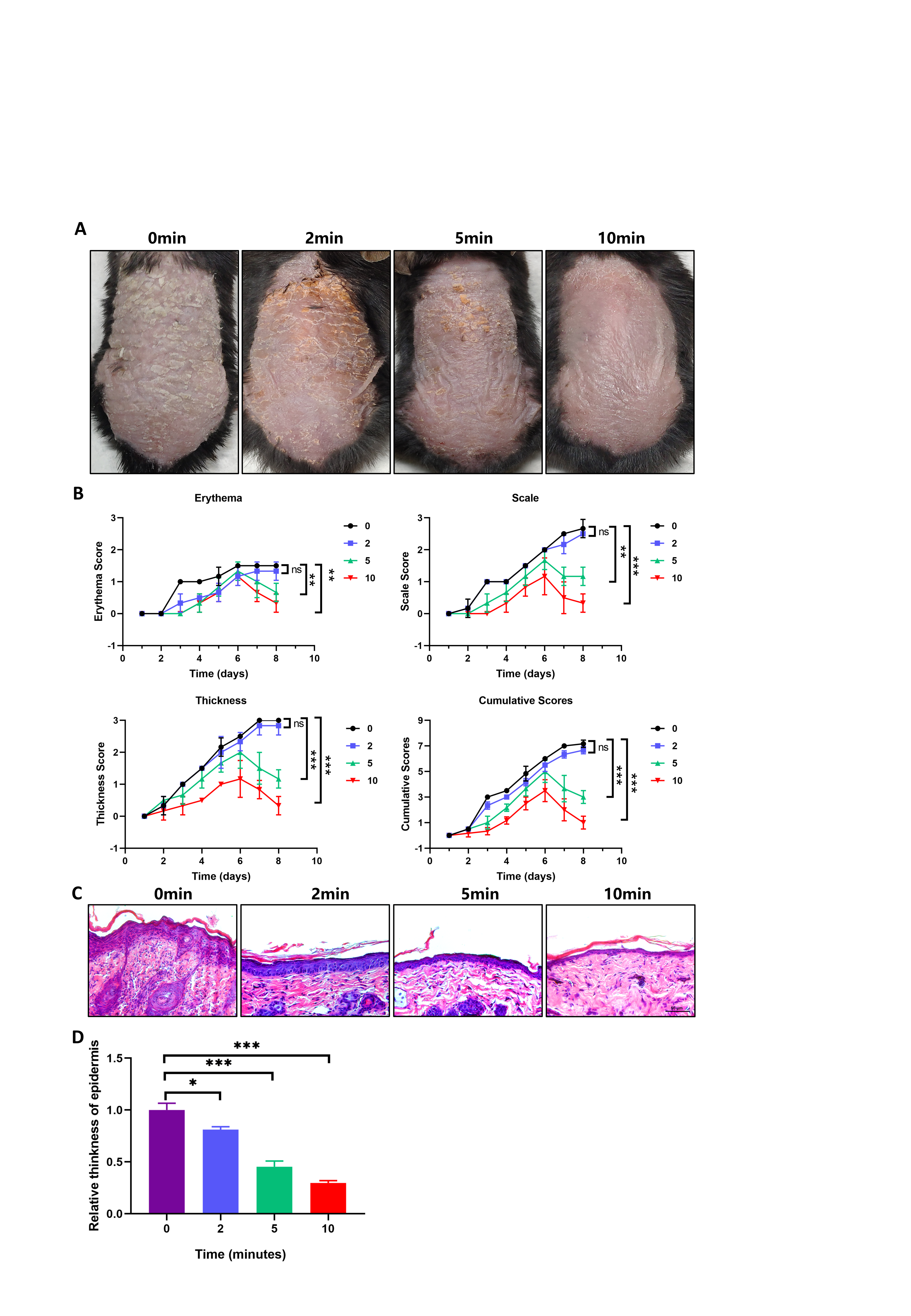


**Figure. S3. Determination of the irradiation time for TBTDC N Ps-PDT in vitro. (A)** Macroscopic appearance of IMQ-induced psoriatic mice treated with TBTDC NP-PDT for seven consecutive days under different irradiation times; **(B)** Erythema, scale, and thickness scores were evaluated daily based on the PASI, and statistical differences among all groups are indicated; **(C)** H&E staining of skin lesions in all the groups. Bar, 50 μm; **(D)** Epidermal thickness of all groups was calculated using ImageJ 1.8.0.


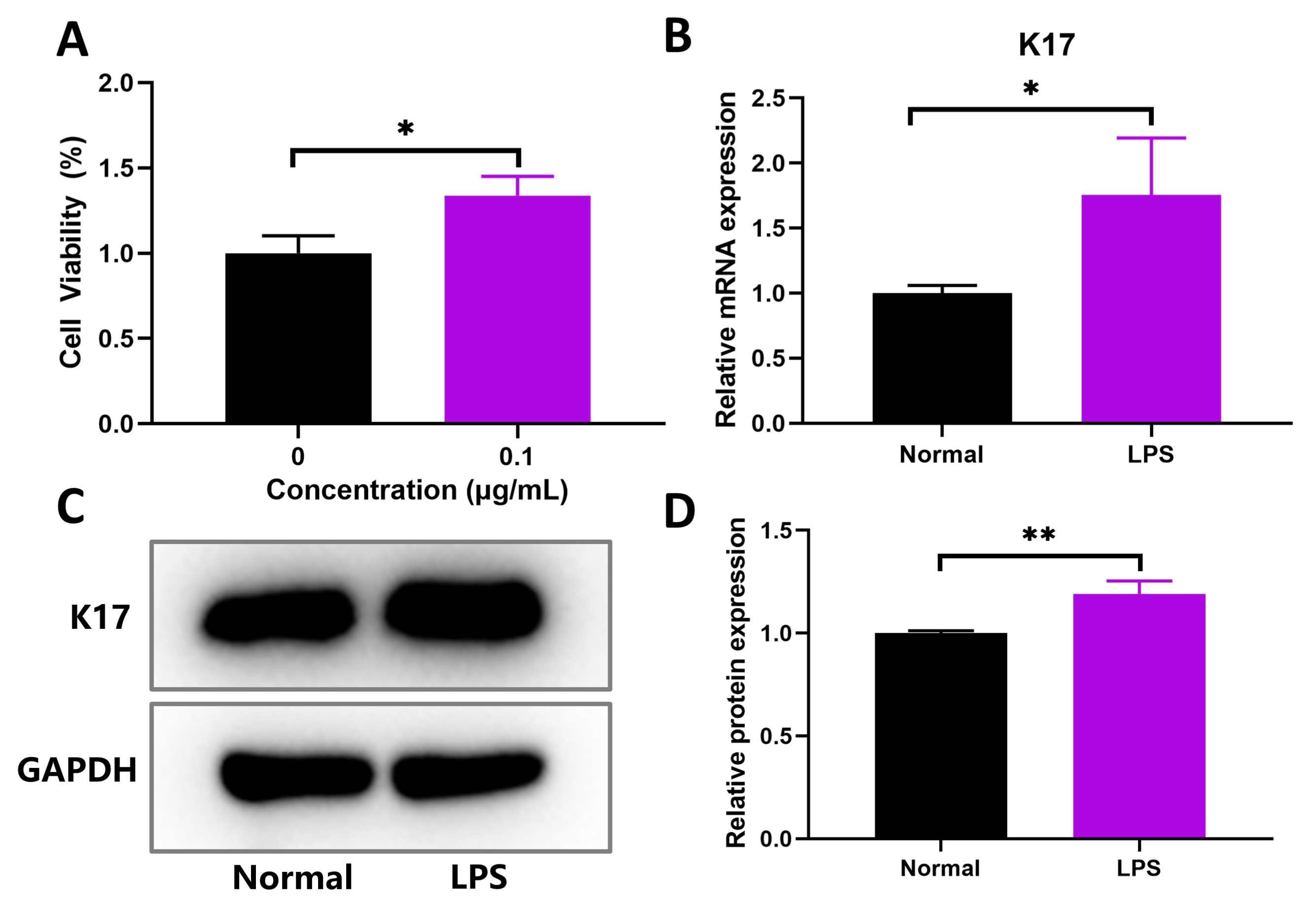


**Figure. S4. Construction of psoriasis cell model in vivo. (A)**Viability of HaCaT cells treated with different concentrations of LPS for 24 h using the CCK-8 method; **(B)** mRNA expression of K17 in HaCaT cells stimulated with LPS for 24 h; **(C)** Protein levels of K17 and **(D)** quantitative analysis of the expression of K17 in HaCaT cells after stimulation with LPS for 24 h.


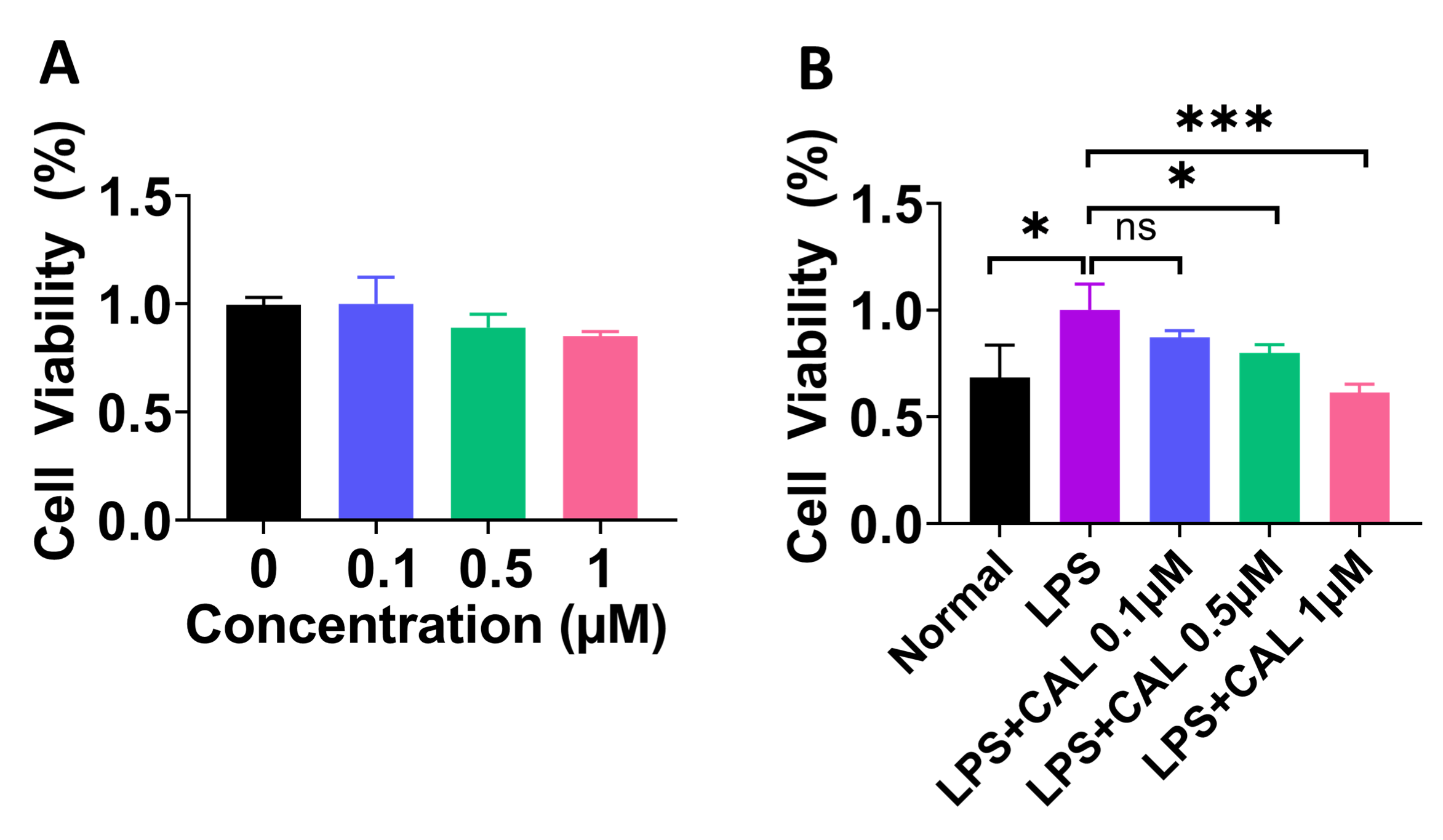


**Figure. S5. Determination of the therapeutic concentration of CAL in vivo. (A)** Viability of HaCaT cells treated with different concentrations of CAL for 24 h using the CCK-8 method; **(B)** Viability of LPS-induced psoriatic keratinocytes treated with different concentrations of CAL for 24 h using the CCK-8 method.


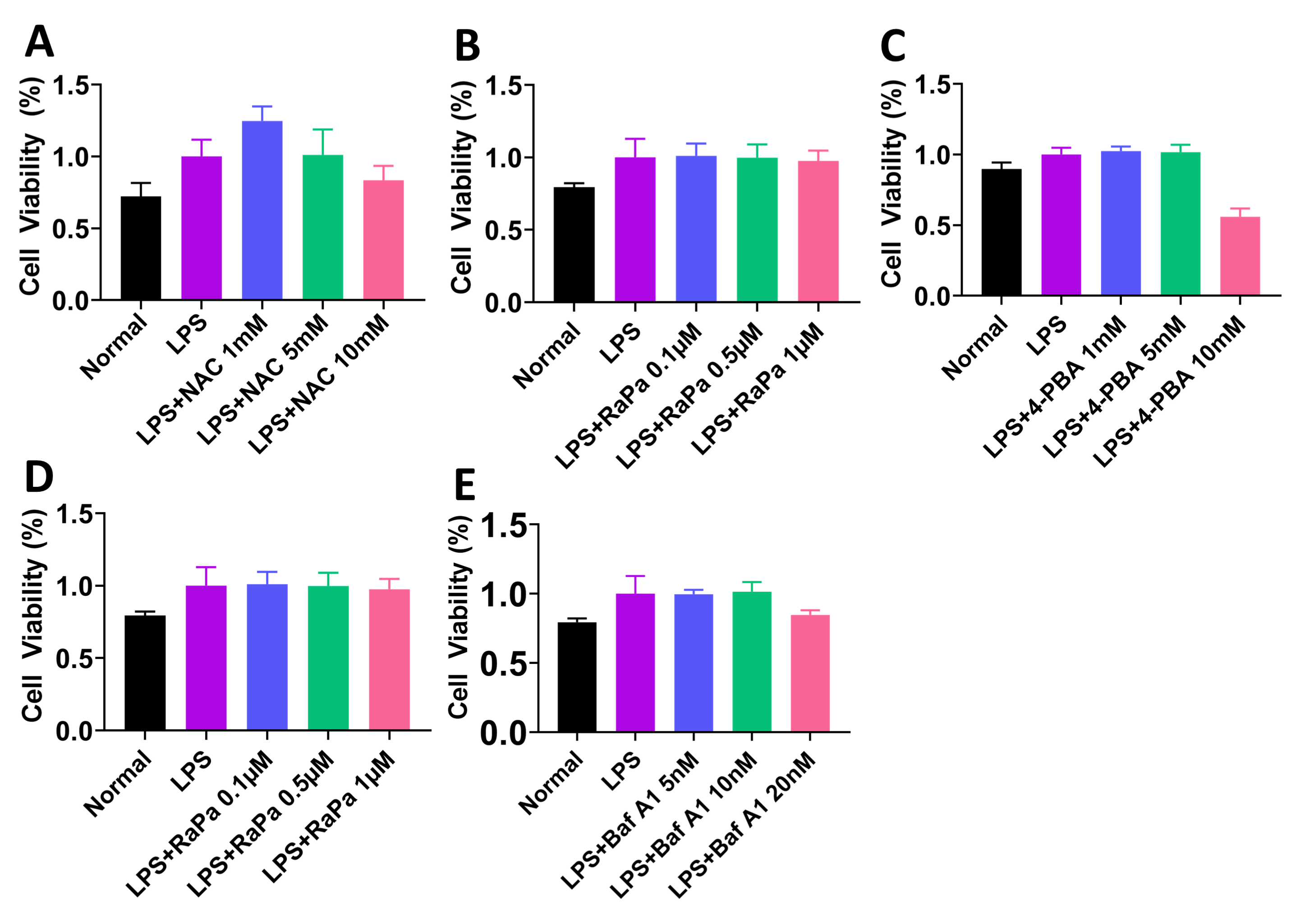


**Figure. S6. Determination of the applied concentrations of inhibitors and activators used in vivo.** Viability of LPS-induced psoriatic keratinocytes treated with different concentrations of **(A)** NAC, **(B)** TM, **(C)** 4-PBA, **(D)** Baf A1, or **(E)** RaPa for 24 h using the CCK-8 method.

| Gene | Forward sequence | Reverse sequence |
| --- | --- | --- |
| β-actin | GGCTGTATTCCCCTCCATCG | CCAGTTGGTAACAATGCCATGT |
| IL-1β | GAAATGCCACCTTTTGACAGTG | TGGATGCTCTCATCAGGACAG |
| IL-17 | TTTAACTCCCTTGGCGCAAAA | CTTTCCCTCCGCATTGACAC |
| IL-23 | GACCCACAAGGACTCAAGGACAA | TGAAGATGTCAGAGTCAAGCAGGTG |
| IL-22 | GGTGACGACCAGAACATCCA | CAGCAGGTCCAGTTCCCCAAT |
| IL-6 | CTGCAAGAGACTTCCATCCAG | AGTGGTATAGACAGGTCTGTTGG |
| TNF-α | CAGGCGGTGCCTATGTCTC | CGATCACCCCGAAGTTCAGTAG |
| IFN-γ | GCCACGGCACAGTCATTGA | TGCTGATGGCCTGATTGTCTT |
| Grp78 | CTGCTGAGGCGTATTTGGGA | TCGCTGGGCATCATTGAAGT |
| CHOP | AATAACAGCCGGAACCTGAGG | CTCATACCAGGCTTCCA GCTC |

**Table S1. Primer sequences targeting genes in mice.**

| Gene | Forward sequence | Reverse sequence |
| --- | --- | --- |
| GAPDH | CACATGGCCTCCAAGGAGTAA | TGAGGGTCTCTCTCTTCCTCTTGT |
| IL-1β | ATGATGGCTTATTACAGTGGCAA | GTCGGAGATTCGTAGCTGGA |
| IL-17A | CGGACTGTGATGGTCAACCTGA | GCACTTTGCCTCCCAGATCACA |
| IL-23A | CTCAGGGACAACAGTCAGTTC | ACAGGGCTATCAGGGAGCA |
| IL-22 | AGGCACTTACTGGCAACAGCA | TGTCTGAGGTTTCACTGGTAAGG |
| IL-6 | ACTCACCTCTTCAGAACGAATTG | CCATCTTTGGAAGGTTCAGGTTG |
| TNF-α | GAGGACCTGGGAGTAGATGAG | GAGGACCTGGGAGTAGATGAG |
| K17 | ACCATGCAGGCCTTGGAGA | GTCTTCACATCCAGCAGGA |
| Grp78 | CTTGCCGTTCAAGGTGGTTG | TCCCAAATAAGCCTCAGCGG |
| CHOP | GCAGCGACAGAGCCAAAATC | TGCTTTCAGGTGTGGTGATGT |

**Table S2. Primer sequences targeting genes in humans.**
